# Combined treatment of benznidazole with an electrolyzed oxidizing water against *Trypanosoma cruzi* on immunological and anatomopathological features in a murine model of Chagas disease

**DOI:** 10.3389/fimmu.2026.1872285

**Published:** 2026-08-25

**Authors:** Olivia Rodríguez-Morales, Alberto Aranda-Fraustro, José Luis Rosales-Encina

**Affiliations:** 1 Laboratory of Molecular Immunology and Proteomics, Department of Molecular Biology, Instituto Nacional de Cardiología Ignacio Chávez, Mexico City, Mexico; 2 Department of Pathology, Instituto Nacional de Cardiología Ignacio Chávez, Mexico City, Mexico; 3 Laboratory of Molecular Biology, Department of Infectomics and Molecular Pathogenesis, Centro de Investigación y de Estudios Avanzados del Instituto Politécnico Nacional, Mexico City, Mexico

**Keywords:** BALB/c mice, benznidazole combination, Chagas disease, electrolyzed oxidizing water, treatment, *Trypanosoma cruzi*

## Abstract

**Background:**

The rising reports of drug resistance in *Trypanosoma cruzi* strains and the high toxicity of current treatments necessitate the exploration of therapeutic alternatives for Chagas disease (ChD). This study evaluated the efficacy of combining benznidazole (BZN) with electrolyzed oxidizing water (EOW) to assess its impact on clinical parameters, immune response, and tissue integrity.

**Methods:**

Forty female BALB/c mice were infected intraperitoneally with 150 blood trypomastigotes of the *T. cruzi* H8 strain. Subjects were divided into eight experimental groups across two independent trials. We monitored parasitemia, weight fluctuations, and survival. Humoral response (IgG and subclasses) and cellular profiles (pro-inflammatory and anti-inflammatory cytokines) were determined via ELISA and flow cytometry, respectively. Histopathological and anatomopathological assessments of target organs were conducted at necropsy.

**Results:**

The BZN+EOW regimen achieved a 99.2% reduction in parasitemia and a 100% survival rate. Although the combination did not statistically surpass BZN monotherapy in absolute parasite clearance, it promoted significant clinical recovery, with weight gain exceeding 50% compared to the untreated infected group. Immunologically, the combination triggered a robust Th1-type immune profile response during the acute phase (40 dpi). By 365 dpi, this response shifted toward a more counterbalanced Th1/Th2 immune microenvironment, correlating with minimal and non-severe histopathological alterations in cardiac and skeletal tissues.

**Discussion:**

These findings suggest that BZN+EOW therapy preserved the trypanocidal efficacy observed with the monotherapy, yet it presented the added advantage of subtly refining the inflammatory landscape in the chronic phase. This balanced immune response could help prevent severe chronic tissue damage, offering a promising adjunct approach to improve the prognosis for ChD.

## 1 Introduction

Chagas disease (ChD) is a chronic condition caused by the protozoan *Trypanosoma cruzi*, which remains a public health issue in Latin America, where 70 million people live in areas at risk of infection and 6 million more are infected with this parasite (1). It is considered one of the most important neglected tropical diseases due to its socioeconomic impact, the work incapacity it causes, and the costs for outpatient care that range between US$4,463 and US$9,601, as well as for the treatment of hospitalized patients in emergency units that are estimated between US$6,700 and US$11,838 annually (2). It is transmitted to humans and various mammals, and in 2022 there was evidence that reptiles and birds are also susceptible to ChD (3, 4).

Treatment for *T. cruzi* infection still relies on drugs approved more than 50 years ago: nifurtimox and benznidazole (BNZ). Their safety and efficacy characteristics are far from ideal and depend primarily on the stage of infection and the age of the patient (5). Research and development of new drugs against *T. cruzi*, with improved safety profiles compared to those currently available, will be essential for the eradication of this endemic parasite that affects millions of people. Both drugs are fully effective in curing the disease if administered early in the acute phase of the infection, including in cases of congenital transmission. However, their efficacy decreases over time, and adverse reactions are more common in older patients (1). The severity of side effects leads to permanent treatment discontinuation in 6% to 40% of people receiving nifurtimox and 7% to 30% of those treated with BNZ (6). The urgent need to find more effective and safer therapeutic alternatives for ChD is evident. In this context, electrolyzed oxidizing water (EOW) has emerged as a promising option. Previous researches have suggested that this solution could have beneficial effects in the treatment of ChD, demonstrating encouraging results in animal models (7, 8).

EOW belongs to the broad group of antiseptics commonly used in medicine, veterinary medicine, and the food industry (9); there are three main types of EOW: acidic, alkaline, and neutral, the latter being used most frequently. They are non-toxic, non-corrosive, and environmentally friendly; therefore, they are used to reduce or eliminate pathogenic and spoilage/altering microorganisms, as well as in hospitals and some research laboratories (10). The efficacy of EOW has been documented in fungi, spores, viruses, and bacteria, including multidrug-resistant strains and biofilms (11). It has been recognized by Japan and South Korea as a new type of drug for the treatment of various intestinal diseases, due to its known efficacy (12). They are produced by the controlled electrolysis of an aqueous sodium chloride solution that generates reactive oxygen species and chlorine, such as hydrogen peroxide and hypochlorous acid (HOCl); these act by capturing electrons and breaking chemical bonds in the outer envelopes of microorganisms. Consequently, it generates protein denaturation in viruses and osmotic lysis in unicellular organisms (9, 10, 13).

This study proposes a combined therapeutic approach of BNZ with an EOW in a murine model infected with *T. cruzi*. The aim of evaluating the efficacy of this combination is not only to improve or match therapeutic outcomes, but also to reduce the adverse effects associated with conventional treatment.

## 2 Methods

### 2.1 Experimental animals

Eighty female BALB/c mice, 6–8 weeks old, were obtained from the Laboratory Animal Production and Experimentation Unit of the Center for Research and Advanced Studies of the National Polytechnic Institute (UPEAL-CINVESTAV). The animals were randomly divided into eight groups with five mice per group (*n* = 5 per cohort): (i) HEALTHY, uninfected mice; (ii) Tc, infected mice without any treatment; (iii) BNZ, uninfected mice treated with benznidazole; (iv) EOW, uninfected mice treated with electrolyzed oxidizing water; (v) BNZ+EOW, uninfected mice treated with the combination of benznidazole and electrolyzed oxidizing water; (vi) Tc/BNZ, infected mice treated with benznidazole; (vii) Tc/EOW, infected mice treated with electrolyzed oxidizing water; and (viii) Tc/BNZ+EOW, infected mice treated with the combination of benznidazole and electrolyzed oxidizing water. To ensure biological reproducibility, all procedures were carried out in two independent experimental blocks phased over time (N = 10 total mice per group) (**Supplementary Figure 1**). Longitudinal clinical parameters, such as parasitemia kinetics and survival rates, are presented as representative data from a single experimental block (n = 5). In contrast, to maximize statistical power and account for infection-induced mortality, data from both independent blocks were pooled (n ≤ 10) for terminal end-point analyses, including net body weight gain/loss, organ indices, cytokine quantification, and histopathological evaluations.

Mice were housed on a 12/12 h light/dark cycle, with a temperature between 22 °C and 24 °C, and provided with food and water *ad libitum*. Animal handling procedures were carried out in accordance with the Mexican Official Standard NOM-062-ZOO-1999: Technical specifications for the production, care, and use of laboratory animals (14), as well as with international guidelines for biomedical research principles involving the use of animals (15).

### 2.2 Infection

The animals were inoculated with 150 parasites (blood trypomastigotes) of the H8 *Trypanosoma cruzi* strain (MHOM/MX/1992/H2 Yucatán (*T. cruzi*) from previously infected mice at peak parasitemia (approximately 21 days post-infection –dpi). Infected blood was collected in a microcentrifuge tube via the caudal vein; parasites were counted in a Neubauer chamber, and the volume was adjusted to infect each mouse with 200 μL intraperitoneally using an insulin syringe with a 27G × 13 mm needle.

### 2.3 Parasitemia and survival monitoring

Circulating blood trypomastigotes (BT) were quantified every other day starting at 10 dpi using a modified Petana technique (16). This monitoring continued until BT counts remained negative for three consecutive observations. Briefly, 10 µL of blood obtained via tail venipuncture was diluted in 490 µL of saline solution (1:50 dilution). A Neubauer chamber was then loaded with a 10 µL aliquot of the suspension to quantify BT within the leukocyte counting quadrants. Additionally, mouse survival was monitored daily throughout the study period (365 dpi) to determine the cumulative mortality rate prior to scheduled euthanasia.

### 2.4 Clinical condition

Each mouse was individually examined from the first day of infection until the day of euthanasia (12 months post-infection –mpi). A score of 1 to 5 was assigned to quantitatively represent clinical status, where 1 represents a healthy clinical status and 5 represents multiple organ failure (**Table 1**).

**Table 1:** Clinical condition scoring scale.

Score	Description
1	No visible signs
2	Mild piloerection
3	Marked piloerection, squinting of the eyes and/or moderate adynamia
4	Closed eyes, hunched spine, marked adynamia, alopecia and/or abdominal distension
5	Cachexia and/or hindlimb paralysis

### 2.5 Body weight recording

Body weight was recorded on a scale (Sartorius^®^, model BL 1500 S, Göttingen, Lower Saxony, Germany) every seven days. This parameter was determined starting at 0 dpi and continued until euthanasia (12 mpi). Data were grouped by phase of infection, in which the weight difference was calculated to determine whether there was weight gain or loss between groups.

### 2.6 Treatments

After ten days of infection, the following treatments were administered orogastrically: 100 μL of 60 ppm EOW (Soluvet^®^, Esteripharma), 100 μL of 1 mg/mL (dose 10 mg/kg) BNZ, or 100 μL of 60 ppm EOW plus 100 μL of 1 mg/mL (dose 10 mg/kg) BNZ twice daily for 30 days. BNZ was prepared using 205.5 mg of the lyophilized N-Benzyl-2-nitro-1H-imidazole-1-acetamide – 97% (Sigma-Aldrich Lab & Production Materials, Missouri, USA), subsequently, 200 mL of 5% Tween 20 were added, obtaining a suspension that was homogenized using a magnetic stirrer (Thermo Fisher Scientific, FisherbrandTM, Pittsburgh, PA, USA) for 30 minutes. The final drug concentration was 0.2055 mg/100 μL.

Orogastric administration was performed with a No. 18 straight stainless-steel feeding cannula (Cadence Science Inc.^®^, Pike, Cranston, RI, USA). In the case of the combined treatment, 100 μL of EOW were administered first and 5 min later the 100 μL of BNZ because EOW is considered safe and mice show better tolerance to oral ingestion of this product compared to BNZ.

### 2.7 Serum samples

Blood was obtained by means of a superficial cut in the skin with a sterile scalpel blade perpendicular to the coccygeal vein. Blood was collected by dripping into microcentrifuge tubes without anticoagulant. The collected volume did not exceed 12.5% ​​of the circulating blood volume (approximately 250 μL per mouse). This procedure was performed at three significant times during the experiment as shown in the timeline of the study (**Supplementary Figure 1**): (i) before infection (day 0), (ii) 3h after the second dose on the last day (day 30) of treatment during the acute phase of ChD (40 dpi), and (iii) in chronic phase of ChD before the euthanasia (365 dpi). Blood samples were kept at room temperature until clot retraction (30 min approximately), centrifuged at 3,500 rpm for 15 min at 4 °C, and aliquoted in 0.6 mL microtubes with 50 µL of serum to be stored frozen al -20 °C until use.

### 2.8 Antibody titration and isotyping

Enzyme-Linked Immunosorbent Assays (ELISAs) were performed to determine the titer and isotypes of the antibodies produced (total IgG, IgG1, and IgG2a) at 40 and 365 dpi. Each serum was tested in duplicate. Lidded 96-well plates (Corning^®^, New York, USA) were sensitized with 1 μg of *T. cruzi* antigen extract in 200 μL of carbonate buffer (NaCO_3_/NaHCO_3_, pH 9.6) and incubated at 37 °C for 1 h. The wells were washed five times with 200 μL of 0.05% Tween 20 in phosphate buffer solution (PBS) in an automatic plate washer (BioTeck^®^, Arcugnano, Italy). Plates were blocked with 200 μL per well of PBS-0.05% Tween 20 (PBS-T) with 5% bovine serum albumin (BSA) (blocking buffer) and incubated at 37 °C for 20 min. The washing step was repeated, 1 μL of mouse serum samples in 200 μL of blocking buffer was added, and the plates were incubated at 37 °C for 1 h. Five washes were performed and 200 μL of the second antibody (Novus Biologicals^®^, Denver, USA) was added per well as follows: horseradish peroxidase (HRP)-labeled anti-total IgG and anti-IgG1 at a dilution of 1:10,000, and HRP-labeled anti-IgG2a at a dilution of 1:5,000 in blocking buffer. The washing step was repeated, 150 μL of the ortho-phenyl diamine (OPD) substrate diluted in citrate buffer at pH 5-0.03% H_2_O_2_ was added and the plates were incubated at room temperature for 10 min; immediately after, the stop solution (50 μL of 5 N H_2_SO_4_) was added and the absorbance was read in a spectrophotometer (Microplate Reader 800 TS BioTeck^®^, Arcugnano, Italy) at 490 nm wavelength. ELISA measurements were performed in duplicate in two independent experiments, the cutoff value was obtained from a negative control serum plus three standard deviations (SD), and a serum from *T. cruzi* chronically infected mouse was used as positive control.

### 2.9 Determination of cytokines in mouse sera

The cytokine profile was evaluated using flow cytometry two distinct time points across two independent experiments. First, to ensure the observation of the compounds’ immune modulatory effect, it was assessed the cytokine profile at 3 h post-treatment on the final day of therapy. This time point was specifically chosen to reflect the reported therapeutic peak of BNZ (relative to its stable serum concentration, *steady-state*), thereby allowing us to capture the shift in immune balance resulting from the reduced pathogen load (17). The second time was upon euthanasia in the chronic stage. Serum levels of nine cytokines: IFN-γ, IL-1β, TNF-α, IL-2, IL-12 and IL-18 of Th1 type immune response, and IL-4, IL-6 and IL-10 of Th2 type immune response were quantified using the LEGENDplex-Custom Mouse 9-plex Panel bead-based immunoassay (BioLegend^®^, San Diego, CA, USA) following the supplier’s instructions as described below: one day prior to the assay, a 10 mL aliquot of the Assay Buffer, the capture bead mix, the standard lyophilized, Matrix C, the 1X Wash Buffer was prepared and the mouse serum samples were aliquoted in three concentrations: concentrated, 1:2 and 1:10. On the day of the assay, the sample reactions and the standard curve were prepared and read on the flow cytometer (BD FACSAria TM Fusion, NJ, USA). A total of 69 serum samples from all experimental groups were analyzed in duplicate. Data obtained from the flow cytometer files were analyzed using BioLegend’s LEGENDplexTM data analysis software available at biolegend.com/en-us/legendplex, and cytokine concentrations were calculated from the standard curve. Cytokine concentrations were presented as the mean values ± standard deviation (SD) of the mean fluorescence intensities (MFI), and were compared across experimental groups. Cytokines data were expressed as MFI values rather than absolute concentrations (pg/mL). This approach was selected to accurately reflect true biological trends and maintain statistical integrity, particularly for cytokines near the lower limits of quantification (LLOQ), thereby avoiding mathematical extrapolation artifacts common in low-concentration ranges.

### 2.10 Macroscopic *post-mortem* examination

Mice were euthanized at 365 dpi by carbon dioxide (CO_2_) asphyxiation in a sealed chamber at a target CO_2_ chamber saturation rate of 70%-90% per minute, in strict compliance with official regulatory standards (NOM-062-ZOO-1999) (14) and institutional Internal Committee for the Care and Use of Laboratory Animals (CICUAL, for its acronym in Spanish) approval. Prior to euthanasia, the body weight of each mouse was recorded, and directed necropsy was performed in order to describe and collect organs of interest such as heart, spleen, lymph nodes, brain, skeletal muscle, esophagus, small intestine, colon, kidneys, and liver. The heart, spleen, and popliteal lymph nodes were weighed to calculate organosomatic indices:


$$Organ\;Index=\frac{organ\;weight}{body\;weight}\times 100$$

Cardiomegaly, splenomegaly, and/or lymphadenomegaly was confirmed when the calculated indices were significantly higher than those observed in healthy control animals.

### 2.11 Histology

The collected organs were washed with saline solution (0.9% NaCl) and then fixed for 48 hours in 10% formalin prepared in saline solution. The fixed organs were washed with deionized water and dehydrated. The organs were sectioned, embedded in paraffin, and histological sections were made. They were stained with hematoxylin and eosin and mounted for microscopic analysis. To ensure maximum objectivity and methodological rigor, all histopathological samples were blindsided and analyzed by a single expert clinical pathologist via a meticulous, systematic examination of more than 5 high-power fields (HPF) per tissue sample. A scoring scale was used to evaluate the degree of tissue damage, with 1 point being the absence of alterations, and as the score progresses, there is a worsening of the histological structures, reaching grade 5 (**Table 2**).

**Table 2:** Histological damage grade score.

Damage grade score	Description
1	Absent = No histological alteration (healthy tissue)
2	Mild = Localized myocarditis/myositis with minimal inflammatory infiltrates
3	Moderate = Multiple foci of myocarditis/myositis (coalescing or diffuse infiltration), localized calcifications, preserved tissue integrity
4	Severe = Pericarditis with generalized myocarditis/myositis, multiple calcifications, localized basophilic necrosis, moderately preserved tissue integrity
5	Very severe/destructive = Pericarditis with generalized myocarditis/myositis, multiple calcifications, generalized basophilic necrosis, tissue integrity completely altered

### 2.12 Statistical analysis

The parasitemia, gain/loss and evolution body weight, antibody and cytokine production assays, as well as the organosomatic indices were averaged and reported with their respective standard deviations (SD) for each experimental group. Data analysis was primarily conducted using Python (for initial processing) and GraphPad Prism version 10 for statistical testing. The distribution of the data was first evaluated using the Shapiro-Wilk normality test. For normally distributed data a one-way analysis of variance (ANOVA) followed by the Tukey *post hoc* test was applied to compare groups; for non-normally distributed data, the Kruskal-Wallis non-parametric test was used. Data derived from physical condition scores and histopathology findings (inflammatory scores in heart tissue) were also analyzed using the Kruskal-Wallis test, followed by the Dunnett’s *post hoc* test for comparison against the control groups. Survival data were specifically analyzed using the Kaplan-Meier method (log-rank test). Statistical significance was primarily defined at *p* ≤ 0.05. However, given the inherent biological variability in *in vivo* models and the exploratory nature of this study, some values *p* ≤ 0.1 were considered to indicate a strong statistical trend. This approach was adopted to avoid Type II errors (false negatives), ensuring that potentially meaningful biological effects of the combined BNZ+EOW therapy –particularly in complex, highly variable immune response assays as isotyping antibodies and cytokines– were not overlooked.

## 3 Results

### 3.1 Establishment of infection and therapeutic response on parasitemia and survival

**Figure 1:**
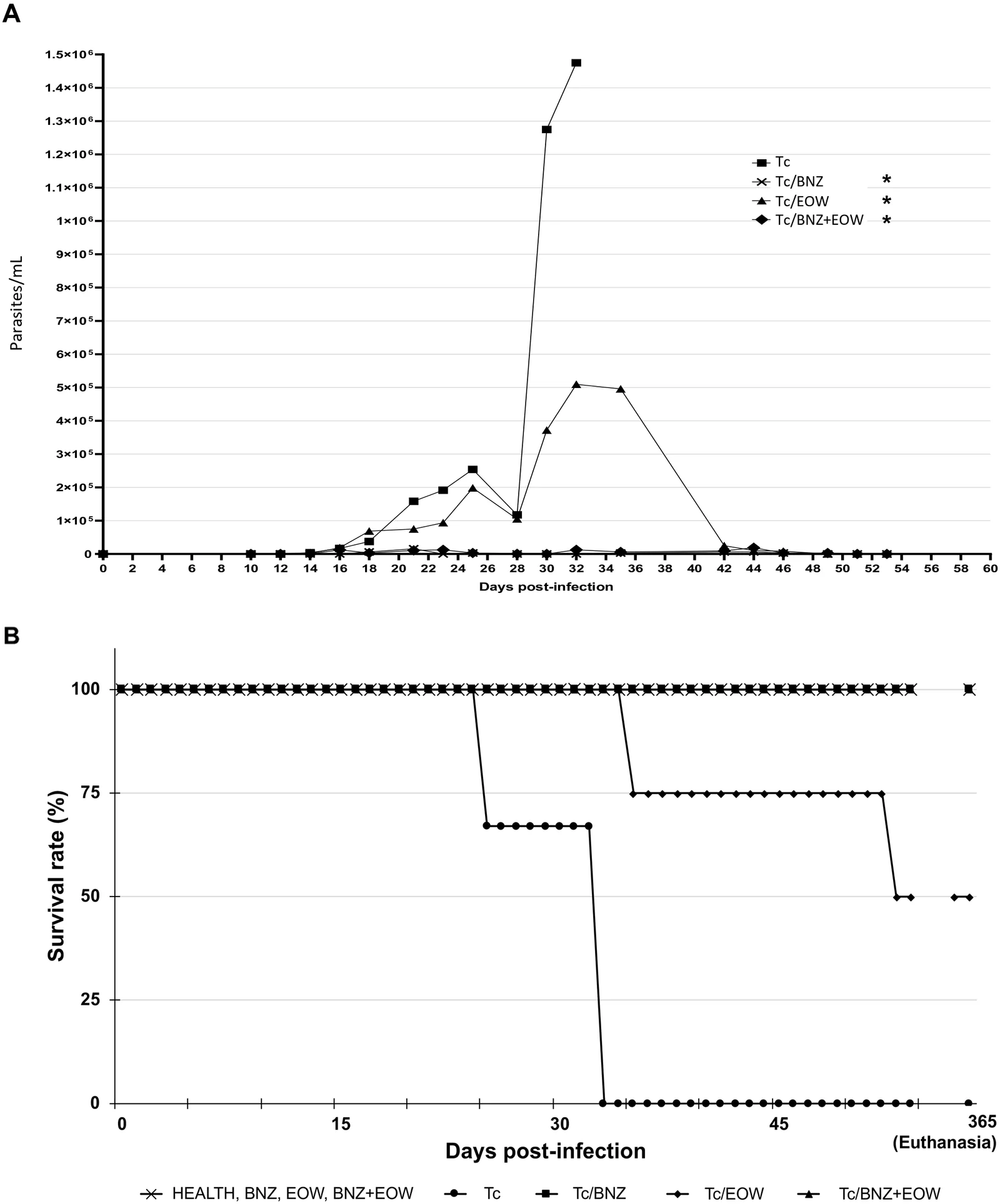
Therapeutic efficacy of combined benznidazole (BNZ) and electrolyzed oxidizing water (EOW) treatment on parasitemia and survival rate. **(A)** Parasitemia curves and **(B)** survival rates of BALB/c mice challenged with 150 blood trypomastigotes of the *T. cruzi* H8 strain. In **(A)**, data points represent the mean parasite load (blood trypomastigotes/mL) from five mice per group (*n* = 5) and are representative of two independent experiments. Statistical significance was determined by two-way ANOVA followed by Dunnett’s *post hoc* test; (*) *p* ≤ 0.05 indicates a significant reduction in parasite load compared to the untreated infected (Tc) group at the peak of parasitemia (day 32). In **(B)**, survival curves correspond to five mice per group (*n* = 5) and are representative of two independent experiments, and were compared between infected/treated groups and their respective controls (HEALTHY, BNZ, EOW and BNZ+EOW). Statistical significance was determined using Kaplan-Meier survival analysis followed by the Log-rank (Mantel-Cox) test; (*) denotes *p* ≤ 0.05. HEALTHY: uninfected/untreated control. Tc: *T. cruzi*-infected control. BNZ and EOW: uninfected groups treated with benznidazole or electrolyzed oxidizing water, respectively. BNZ+EOW: uninfected group treated with the combination. Tc/BNZ, Tc/EOW, and Tc/BNZ+EOW: infected groups treated with benznidazole, electrolyzed oxidizing water, or the combination, respectively.

Infection was confirmed in Groups Tc, Tc/BNZ, Tc/EOW and Tc/BNZ+EOW by monitoring the parasitemia levels starting at 10 dpi. Monitoring continued until three consecutive negative blood counting were obtained. As shown in **Figure 1A** a progressive increase in parasitemia was observed across all groups from 10 dpi. Infected/untreated group (Group Tc) exhibited the highest parasite load, peaking at 28 dpi, which resulted in 100% mortality by day 32. Tc/EOW group showed a 65.5% reduction (*p* ≤ 0.5) in parasitemia compared to the Tc group, whereas the Tc/BNZ and Tc/BNZ+EOW groups reached a near-total reduction of 99% (*p* ≤ 0.05 relative to Tc). The latter two groups displayed comparable and effective control of the parasite load, maintaining significantly lower levels than groups Tc and Tc/EOW throughout the acute phase. These findings demonstrated that the combined administration of BNZ+EOW is as effective as BNZ monotherapy in controlling parasitemia and mortality during the acute phase.

**Figure 1B** shows the survival rate of the experimental groups, comparing the healthy groups with those infected with *T. cruzi.* The healthy groups (Healthy, BNZ, EOW, and BNZ+EOW) maintained 100% survival throughout the experiment. In contrast, the infected groups showed differences in survival depending on the treatment received: Group Tc (untreated infected) showed a rapid decline in survival starting at 27 dpi, reaching 100% mortality by day 32. Although Group Tc/EOW showed greater initial resistance compared to the Tc group, it also experienced a decrease in survival after the completion of EOW treatment, with two deaths recorded starting at 36 dpi, resulting in a 50% survival rate. Groups Tc/BNZ and Tc/BNZ+EOW had significantly higher survival compared to the Tc and Tc/EOW groups, remaining stable throughout the experiment with 100% survival.

### 3.2 Gain/loss and evolution of body weight throughout the experimental animals’ lifetime

**Figure 2:**
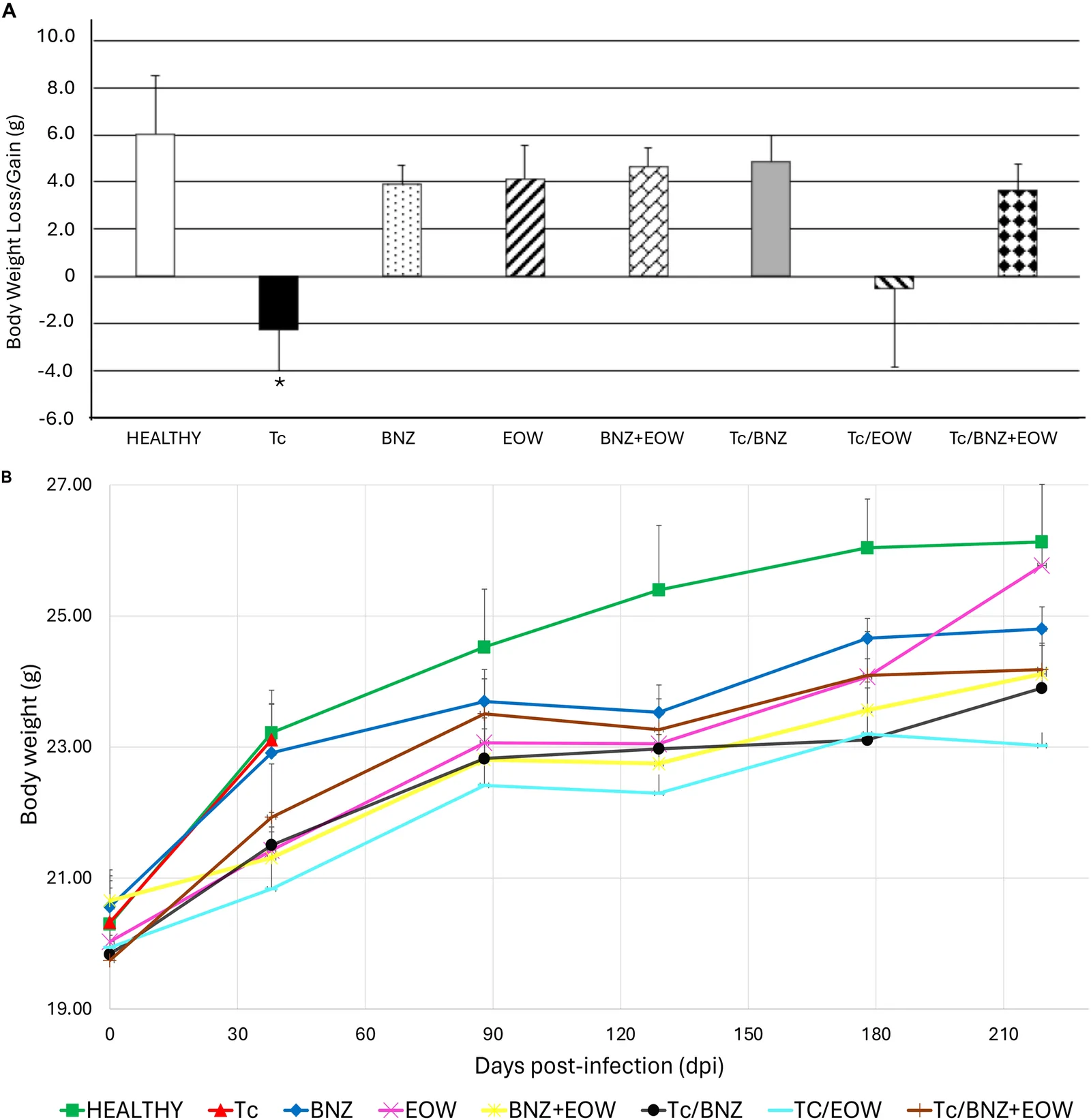
Body weight dynamics in infected and treated BALB/c mice. Mice were either uninfected or infected with *T. cruzi* and administered combined benznidazole and electrolyzed oxidizing water therapy. **(A)** Net weight gain or loss, calculated as the mean difference between terminal weight (at euthanasia at 365 dpi, or at the time of premature mortality due to disease progression) and the initial weight. **(B)** Longitudinal evolution of body weight throughout the study, which corresponds to one representative of two independent experiments. The curve for the Tc group terminates at day 32 due to 100% mortality in this cohort. From day 54 onward, the curve for the Tc/EOW group exclusively represents the remaining 50% surviving populations. Values represent the mean ± SD. Statistical significance was determined by one-way ANOVA followed by Dunnett’s *post hoc* test; (*) *p* ≤ 0.05 indicates a significant difference relative to the HEALTHY group. HEALTHY: uninfected/untreated control. Tc: *T. cruzi*-infected control. BNZ and EOW: uninfected groups treated with benznidazole or electrolyzed oxidizing water, respectively. BNZ+EOW: uninfected group treated with the combination. Tc/BNZ, Tc/EOW, and Tc/BNZ+EOW: infected groups treated with benznidazole, electrolyzed oxidizing water, or the combination, respectively.

**Figure 2A** shows the changes in body weight in the different experimental groups throughout the study. The Group HEALTHY showed the greatest weight gain (6 g). In contrast, for the untreated infected group (Tc), whose record is limited to the acute phase due to 100% mortality by day 32, surviving animals exhibited an average weight loss of 2.2 g, showing a substantial weight reduction associated with the untreated infection. The three groups that only received treatment showed average body weight gains between 3.9 g and 4.6 g, indicating a moderate impact. In the infected and treated groups, body weight gain was observed only in the Tc/BNZ group (4.8 g) and the Tc/BNZ+EOW group (3.6 g), indicating that these two treatment regimens mitigated the negative effects of the infection; however, body weight gain was also affected by the administered therapy, as they did not reach the weight of the animals in the healthy group. The Tc/EOW group showed a body weight loss of 1 g. This value reflects the compiled data from this group, including both the final weights recorded at the end of the study (365 dpi) for the survivors, and the terminal weights of the animas that died to disease progression (representing 50% of the population, with a median survival of 54 dpi).

**Figure 2B** shows the body weight evolution for the different experimental groups. The HEALTHY group showed positive evolution over time, as the individuals in this group gained weight steadily. Mice in the infected groups (with treatment) showed slower weight gain, registering a decrease between 90 and 120 dpi, suggesting that the infection has a detrimental effect on weight gain. Likewise, during the acute phase of the infection (up to day 32), animals treated with BNZ and EOW (either as monotherapies or in combination) showed lower body weight values ​​than the untreated infected animals. Beyond this point, and following the 100% mortality of the Tc group, the treated animals continued to display reduced weight gain compared to the healthy control group. This trend was most evident —though not statistically significant— in the Tc/EOW group, where from 54 dpi onward, body weight records were restricted to the 50% surviving cohort of that group. This pattern suggests that both products may influence weight dynamics, possibly due to transient side effects such as reduced appetite or digestive alterations associated with their administration.

### 3.3 General physical condition

**Figure 3:**
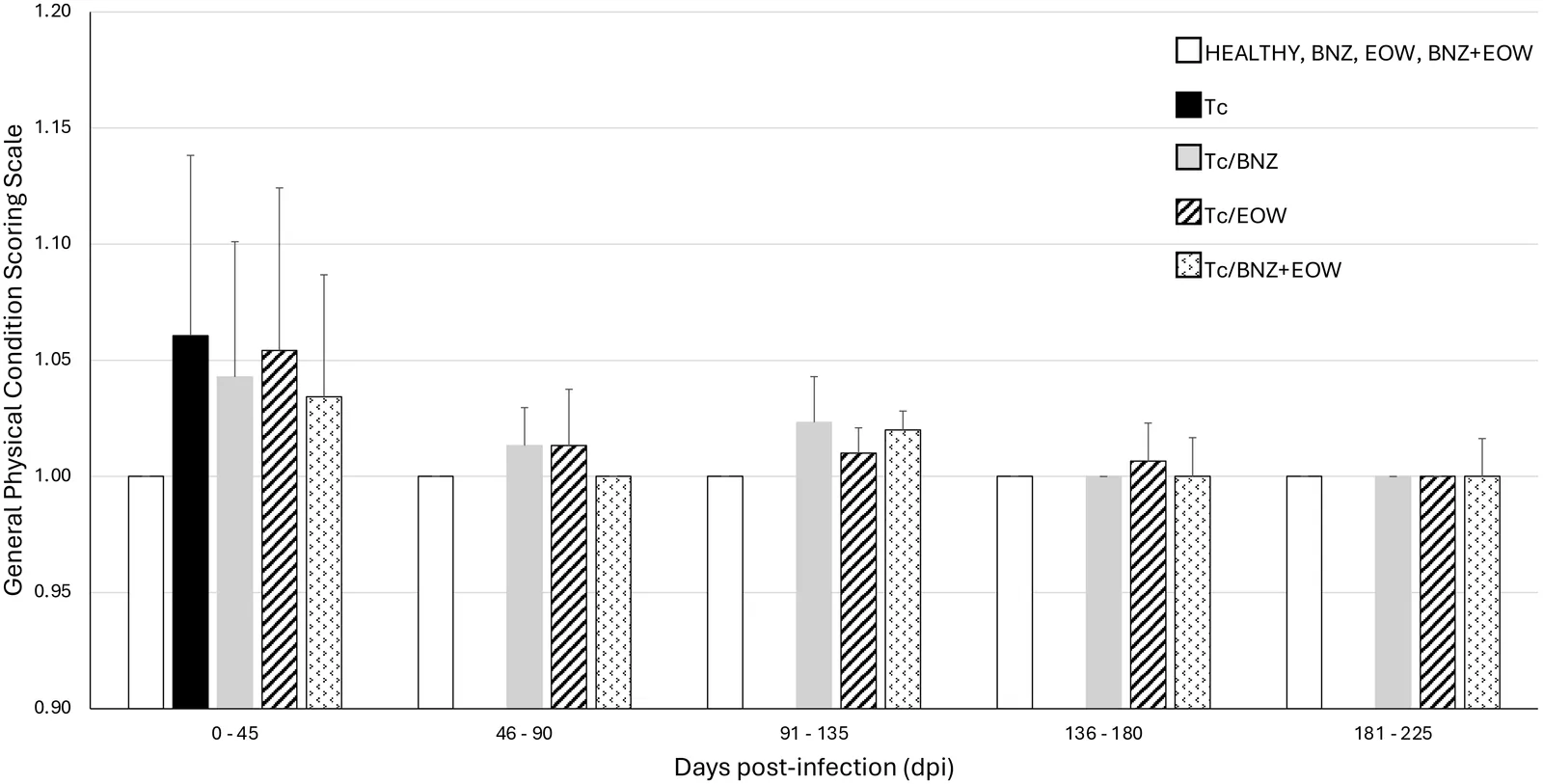
Longitudinal assessment of physical condition in BALB/c mice. Evaluation was performed on uninfected mice and those infected with *T. cruzi* under combined BNZ+EOW therapy from days 11 to 56 post-infection. Data are presented as the mean of assigned scores ± SD per group. Statistical analysis was conducted using the Kruskal-Wallis test followed by Dunn’s *post-hoc* test. HEALTHY: uninfected/untreated control. Tc: *T. cruzi*-infected control. BNZ and EOW: uninfected groups treated with benznidazole or electrolyzed oxidizing water, respectively. BNZ+EOW: uninfected group treated with the combination. Tc/BNZ, Tc/EOW, and Tc/BNZ+EOW: infected groups treated with benznidazole, electrolyzed oxidizing water, or the combination, respectively.

The evolution of general physical condition was assessed using a scoring scale corresponding to the observation of clinical signs of disease (see **Table 1**). The infected groups, with and without treatment, showed higher scores between 16 and 46 dpi, a period corresponding to the acute stage of the disease when parasitemia is detected. The groups that reached the highest levels were the Tc and Tc/EOW groups at 23 dpi. At 32 dpi, mortality rate in the Tc group reached 100%, and it was no longer possible to continue monitoring their overall physical condition. Finally, mice in the remaining groups recovered their baseline score of 1 over time, although the last to do so were the survivors (50%) from the Tc/EOW group (**Figure 3**). Although animals from Tc/BNZ and Tc/BNZ+EOW experienced a brief period of deterioration, they recovered more quickly and regained a physical appearance comparable to that of healthy controls.

### 3.4 Humoral immune response to combined benznidazole and electrolyzed oxidizing water

**Figure 4:**
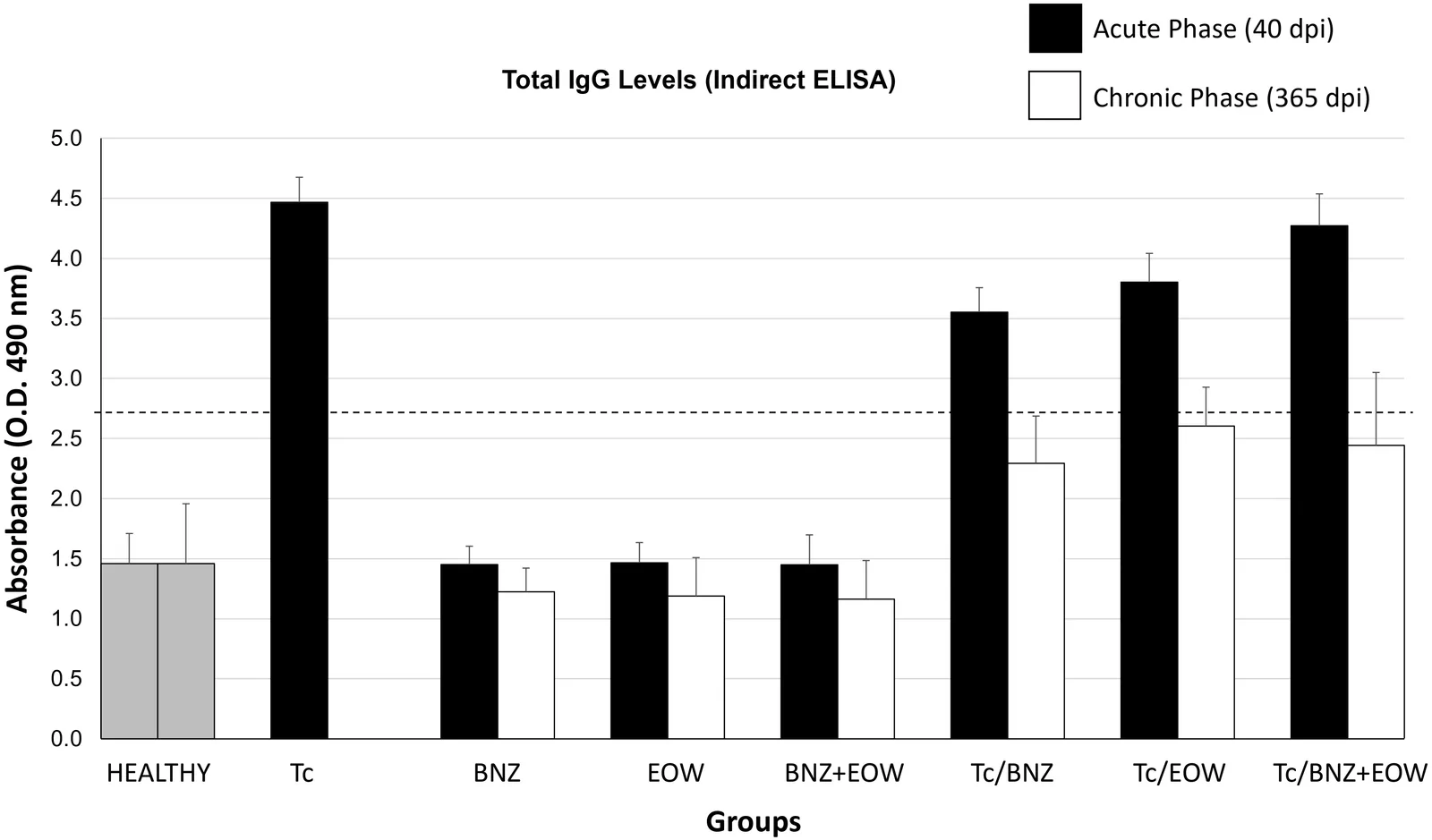
Total anti-*T. cruzi* IgG levels in BALB/c mice during acute and chronic infection. Mice were treated with a combination of BNZ+EOW. Each point represents the mean ± SD (*n* = 5 per group) and is representative of two independent experiments. The horizontal dashed line indicates the cutoff value (CV = 2.273). Black and white bars represent acute and chronic stages, respectively. HEALTHY: uninfected/untreated control. Tc: *T. cruzi*-infected control. BNZ and EOW: uninfected groups treated with benznidazole or electrolyzed oxidizing water, respectively. BNZ+EOW: uninfected group treated with the combination. Tc/BNZ, Tc/EOW, and Tc/BNZ+EOW: infected groups treated with benznidazole, electrolyzed oxidizing water, or the combination, respectively.

The determination of anti-*T. cruzi* IgG antibody levels 3 h after the end of treatment (with a 30-day regimen, twice daily via orogastric route) and at euthanasia in the chronic stage (365 dpi) showed that in all mice infected with *T. cruzi* with and without treatment there was efficient antibody production during the acute stage, which indicates that infection with the parasite stimulated the adaptive immune system of the animals at 40 dpi (**Figure 4**). In contrast, in the chronic stage, antibody levels were on average below the cutoff line, indicating that the groups showed negative seroconversion, which is strongly suggestive of a conventional parasitological cure because the antigen that stimulates the adaptive immune system for IgG production is no longer present; however, the animals must be considered individually, since in each group the number of seronegative individuals was variable: 5/5 (100%) for the Tc/BNZ Group, 3/5 (60%) for the Tc/BNZ+EOW Group and 2/5 (40%) for the Tc/EOW Group, which suggests that although the EOW has a beneficial effect in achieving a conventional pharmacological cure for some animals, the combined therapy of this solution with BNZ is not as effective as the canonical drug alone, and even less effective if used only as monotherapy (**Figure 4**).

**Figure 5:**
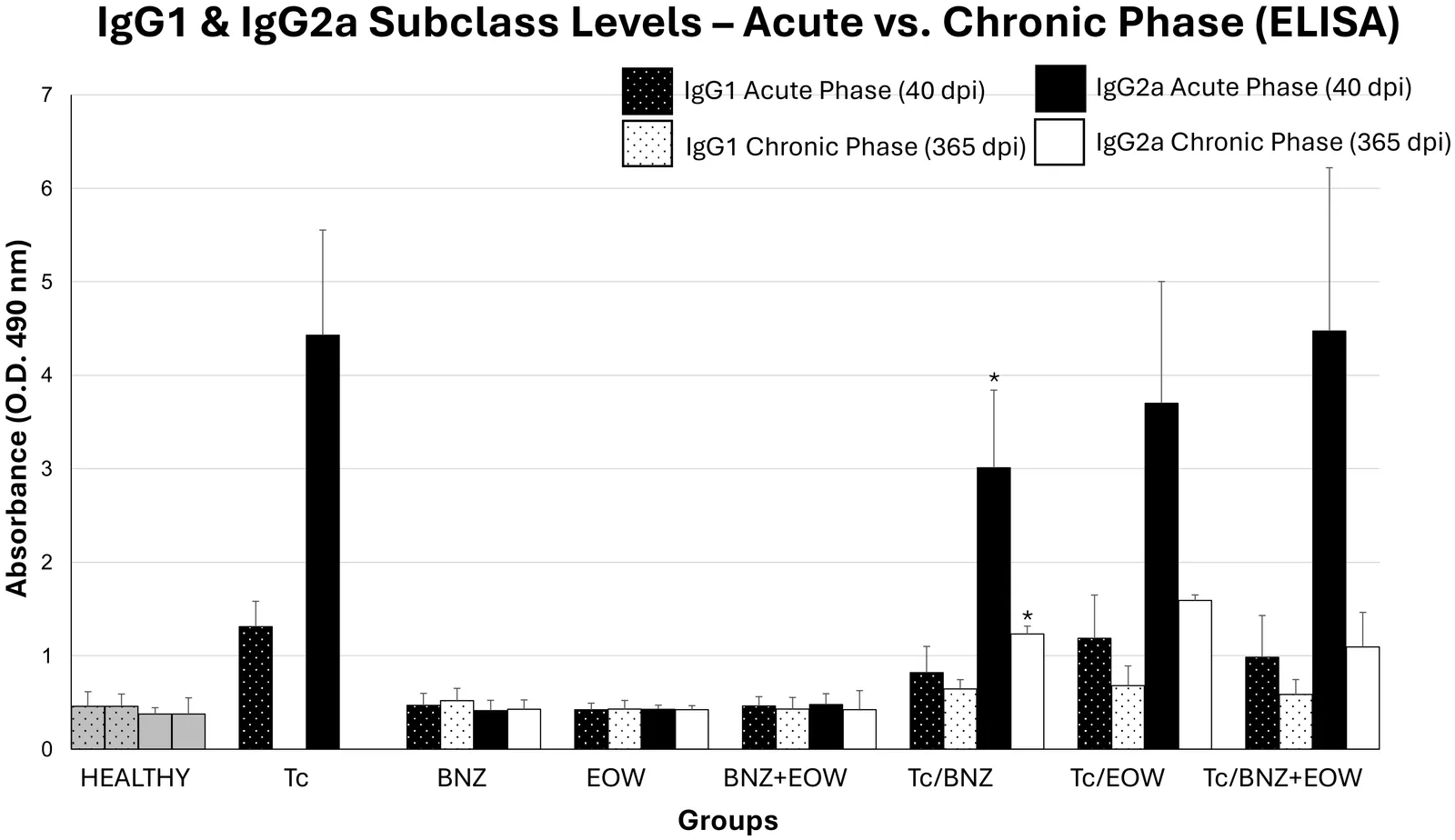
Serum levels of anti-*T. cruzi* IgG1 and IgG2a in infected BALB/c mice. Mice were treated with a combination of BNZ+EOW via the 30-day orogastric regimen. Samples were collected 3 h post-treatment completion during the acute phase and at 365 dpi upon euthanasia in the chronic phase. Bars colors indicate the study phase (black: acute; white: chronic), while patterns denote IgG subclasses (dotted: IgG1; solid: IgG2a). Data represent the mean ± SD of each group and are representative of two independent experiments. Statistical significance between infected and treated groups or healthy and treated groups in acute and chronic phase, respectively, was determined by one-way ANOVA followed by Tukey's *post hoc* test (*p ≤ 0.1). HEALTHY: uninfected/untreated control. Tc: *T. cruzi*-infected control. BNZ and EOW: uninfected groups treated with benznidazole or electrolyzed oxidizing water, respectively. BNZ+EOW: uninfected group treated with the combination. Tc/BNZ, Tc/EOW, and Tc/BNZ+EOW: infected groups treated with benznidazole, electrolyzed oxidizing water, or the combination, respectively.

To evaluate the adaptive immune response established in mice under different treatment regimens, humoral immunity was assessed by measuring serum IgG1 and IgG2a subclasses. Samples were collected three hours after completing the 30-day orogastric administration protocol for the acute phase (40 dpi). The IgG2a/IgG1 ratio increased by 3.4-, 3.7-, and 3.1-fold in the Tc, Tc/BNZ, and Tc/EOW groups, respectively, whereas the Tc/BNZ+EOW group reached a 4.5-fold increase. Statistical analysis revealed that only the Group Tc/BNZ controlled the high production of IgG2a relative to IgG1. Consequently, the combined treatment triggers a marked polarization toward a Th1 pro-inflammatory response. This effect is likely driven by the differential exposure of circulating parasites compared to canonical treatment or infection alone (**Figure 5**, dotted-black and solid-black bars).

Analysis of serum IgG1 and IgG2a subclasses during the chronic stage of the disease revealed a modest increase in IgG2a titers across all treated *T. cruzi*-infected groups. This pattern suggests that the Th1-type pro-inflammatory microenvironment was less pronounced than in the acute phase. This stabilization was particularly evident in the Tc/BNZ and Tc/BNZ+EOW groups, where IgG2a/IgG1 ratios increased by 1.9- and 1.8-fold, respectively. These results likely reflect a comparable efficacy between the canonical and combined treatments in modulating parasite load and promoting a more proportional Th1/Th2 immune profile during the chronic stage. While the Tc/BNZ group showed significant differences in IgG2a levels compared to healthy controls, the lack of statistical significance in the Tc/EOW and Tc/BNZ+EOW groups—despite similar biological trends—may be attributed to the limited sample size (*n* = 2) and high standard deviation, respectively (**Figure 5**, dotted-white and solid white bars).

### 3.5 Combined benznidazole and electrolyzed oxidizing water therapy on systemic cytokine profiles and Th1/Th2 immune response

**Figure 6:**
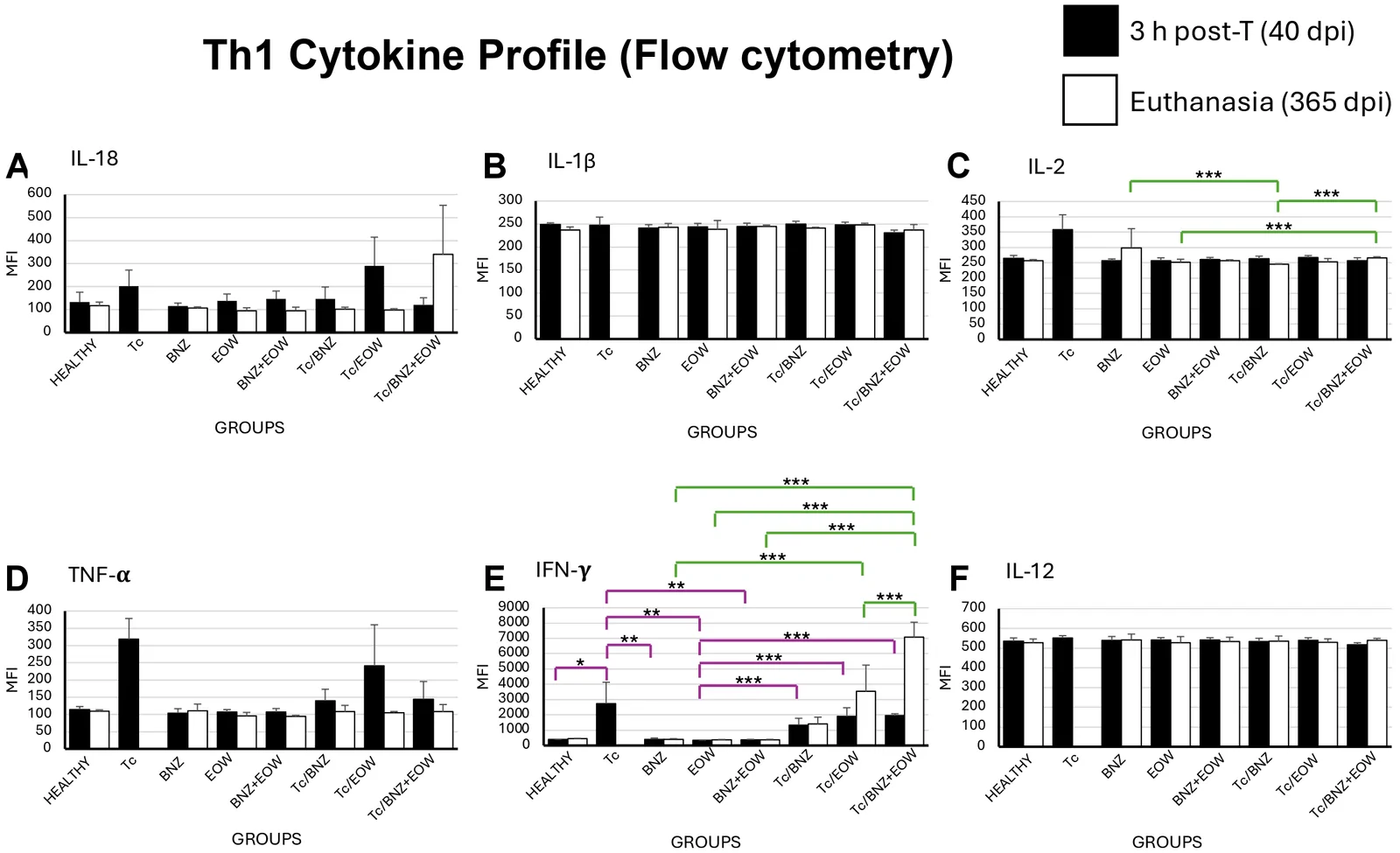
Serum Th1 cytokine profiles in *T. cruzi*-infected BALB/c mice treated with benznidazole (BNZ) and electrolyzed oxidizing water (EOW). Panels show **(A)** IL-18, **(B)** IL-1β, **(C)** IL-2, **(D)** TNF-α, **(E)** IFN-γ, and **(F)** IL-12 levels. A 30-day treatment regimen was initiated at 10 dpi. Samples were collected during the acute phase (3 h post-treatment; black bars) and the chronic phase (upon euthanasia at 365 dpi; white bars). Data are expressed as mean ± SD of the mean fluorescence intensities (MFI), representing the relative protein expression levels. Statistical significance was determined by one-way ANOVA (IL-1β, IL-12) or Kruskal-Wallis test (IL-18, IL-2, TNF-α, IFN-γ). Significance was defined as *p* ≤ 0.1, (*) comparison vs. HEALTHY group; (**) comparison vs. Tc group, and (***) intra-group comparison between treated cohorts. HEALTHY (uninfected/untreated); Tc (infected/untreated); BNZ, EOW and BNZ+EOW (uninfected mice receiving the respective therapies); Tc/BNZ, Tc/EOW, and Tc/BNZ+EOW (infected mice receiving the respective therapies).

The **Figure 6** illustrates the Th1-related cytokine profiles, expressed as Mean Fluorescence Intensity (MFI). Data are presented as MFI values to accurately reflect biological trends, particularly for cytokines that approached the lower limits of quantification in absolute concentration assays.

In the acute phase (3 h post-treatment), IL-18 levels (**Figure 6A**) exhibited moderate variation, with the Tc/EOW group showing the most pronounced increase. Conversely, at 365 dpi, the Tc/BNZ+EOW group reached the highest IL-18 levels, albeit with high inter-individual variability (SD). IL-1β levels (**Figure 6B**) remained uniform across all experimental groups and time points. Regarding IL-2 in the chronic phase (**Figure 6C**), intra-group non-parametric statistical analysis revealed subtle differences among the treated cohorts (*p ≤*0.1). Despite absolute MFI values appearing visually comparable across groups due to minimal data dispersion, a slight downward trend in IL-2 levels was observed in the BNZ monotherapy group, whereas the BNZ+EOW combination showed a slight upward tendency. In **Figures 6D**, TNF-α levels peaked in the Tc group at 3 h post-treatment. Although a non-significant visual elevation was also observed in the Tc/EOW and Tc/BNZ+EOW groups, these variations did not achieve statistical significance (*p* ≤ 0.1) due to high intra-group standard deviations, whereas Tc/BNZ maintained levels closest to baseline like the rest of the other groups. Uninfected control groups receiving mono- or combined therapies maintained baseline levels of IFN-γ (**Figure 6E**). In contrast, *T. cruzi* infection, particularly when coupled with therapeutic interventions, significantly triggered systemic IFN-*γ* production across both acute and chronic phases (***). Notably, the highest elevation was recorded in the Tc/BNZ+EOW group in chronic phase, which exhibited significantly higher MFI levels compared to both the untreated infected cohort (Tc) and the respective monotherapies (Tc/BNZ and Tc/EOW). While infection alone (Tc) during acute phase elicited a moderate inflammatory IFN-*γ* response (~2,700 MFI), its magnitude reached approximately half of the levels observed in the combined treatment group (Tc/BNZ+EOW, ~7,000 MFI) in chronic phase, demonstrating a robust, disease-state-dependent immunomodulatory effect of the dual therapy. Finally, IL-12 levels (**Figure 6F**) were homogeneous throughout the study, with no significant differences observed between treatment regimens or time points.

**Figure 7:**
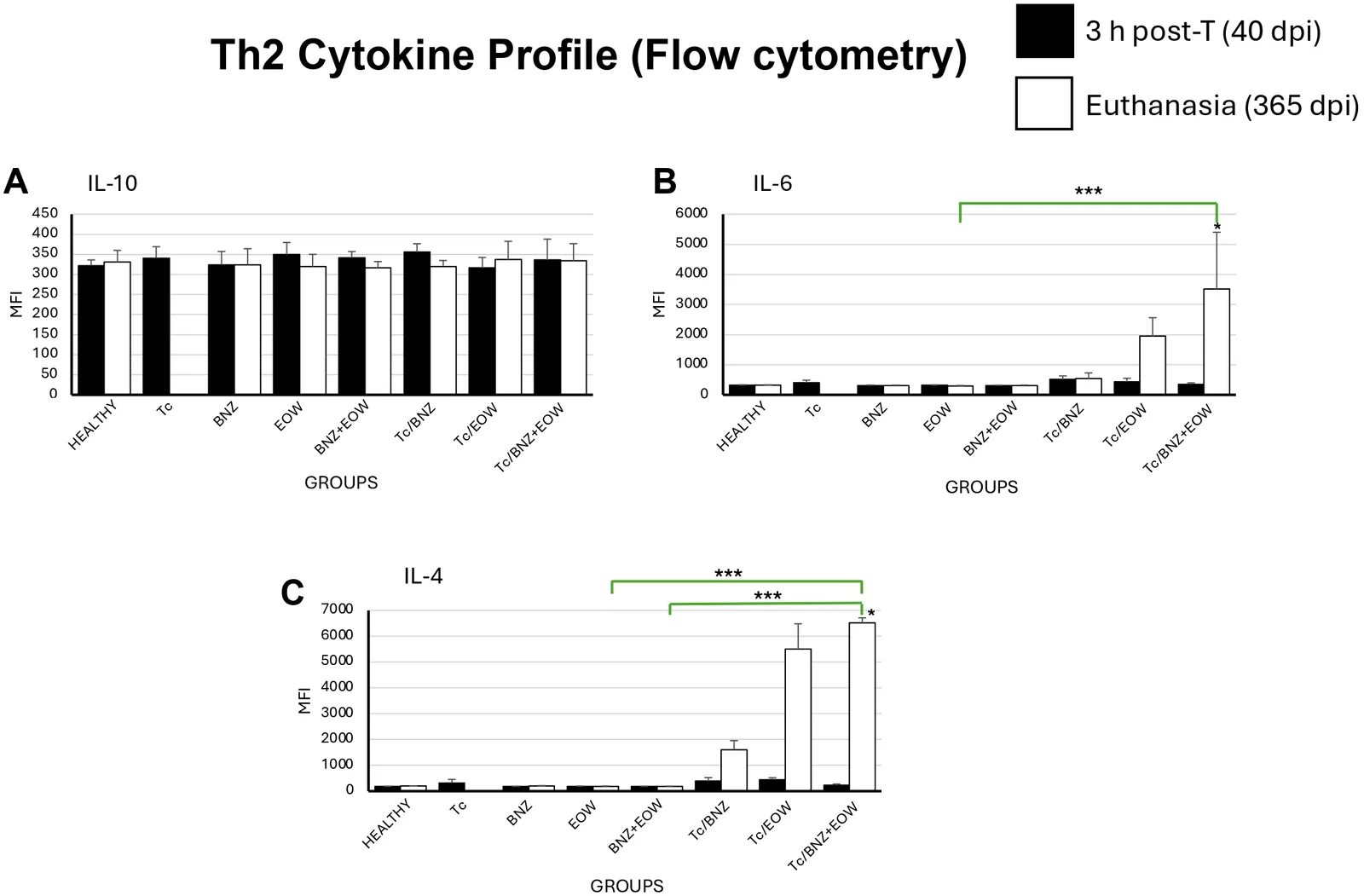
Serum Th2 cytokine profiles in *T. cruzi*-infected BALB/c mice treated with benznidazole (BNZ) and electrolyzed oxidizing water (EOW). Panels display **(A)** IL-10, **(B)** IL-6, and **(C)** IL-4 levels. Treatment was administered for 30 days starting at 10 dpi. Samples were collected during the acute phase (3 h post-treatment; black bars) and the chronic phase (upon euthanasia at 365 dpi; white bars). Bars represent the mean ± SD of the mean fluorescence intensities (MFI), indicating relative protein expression. Statistical significance was determined by one-way ANOVA (IL-10) or Kruskal-Wallis test (IL-4, IL-6), with significance defined as *p* ≤ 0.1 (*) comparison vs. HEALTHY and (**) Tc groups; (***) Comparison between therapy in uninfected groups and the BNZ and EOW combination therapy in infected groups. HEALTHY (uninfected/untreated); Tc (infected/untreated); BNZ, EOW and BNZ+EOW (uninfected mice receiving the respective therapies); Tc/BNZ, Tc/EOW, and Tc/BNZ+EOW (infected mice receiving the respective therapies).

**Figure 7** illustrates the profiles of Th2-associated cytokines, expressed as MFI. Regarding IL-10 (**Figure 7A**), levels remained stable and within comparable ranges across all experimental groups and both time points. In contrast, IL-6 levels (**Figure 7B**) maintained baseline levels during the acute phase across all groups. However, during the chronic phase, a marked elevation was observed specifically in the cohorts infected with *T. cruzi* that received EOW, either as a monotherapy (Tc/EOW) or in combination (Tc/BNZ+EOW). Notably, this chronic modulation reached statistical significance (*p* ≤ 0.1) when comparing the combined therapy cohort (Tc/BNZ+EOW) against its corresponding uninfected control group (EOW). Untreated infected controls (Tc) could not be evaluated at this chronic timepoint due to 100% acute mortality. Finally, in contrast to the baseline values maintained across all groups during the acute phase, IL-4 quantification (**Figure 7C**) revealed an overall increase specifically during the chronic phase across all infected and treated groups. The most pronounced elevation in IL-4 was observed in the two groups receiving EOW (Tc/EOW and Tc/BNZ+EOW), suggesting a specific immunomodulatory effect of this compound on the Th2 response. Notably, the combination therapy (Tc/BNZ+EOW) induced a significantly higher elevation of IL-6 and IL-4 compared to EOW monotherapy (*p* ≤ 0.1), suggesting a synergistic effect between the two compounds.

The IFN-γ/IL-4 ratio was also calculated to characterize the polarization of the immune response across treatment groups (**Supplementary Data**). In the chronic phase, the Tc/BNZ, Tc/EOW and Tc/BNZ+EOW groups exhibited ratios near unity (0.87, 0.64, and 1.08, respectively), suggesting an immune homeostasis that aligns with the previously observed IgG1/IgG2a profiles. However, during acute infection, the Tc/BNZ+EOW group displayed a robust Th1-type polarization, mirroring the untreated infected cohort (Group Tc) and coinciding with the period of increased lethality. Notably, monotherapies with BNZ or EOW attenuated this acute pro-inflammatory surge, achieving a 2.5- and 2-fold reduction in polarization compared to the combined treatment, thereby suggesting a regulatory role in the early stages of infection.

### 3.6 Macroscopic alterations

#### 3.6.1 Cardiomegaly, splenomegaly and lymphadenopathy

**Figure 8:**
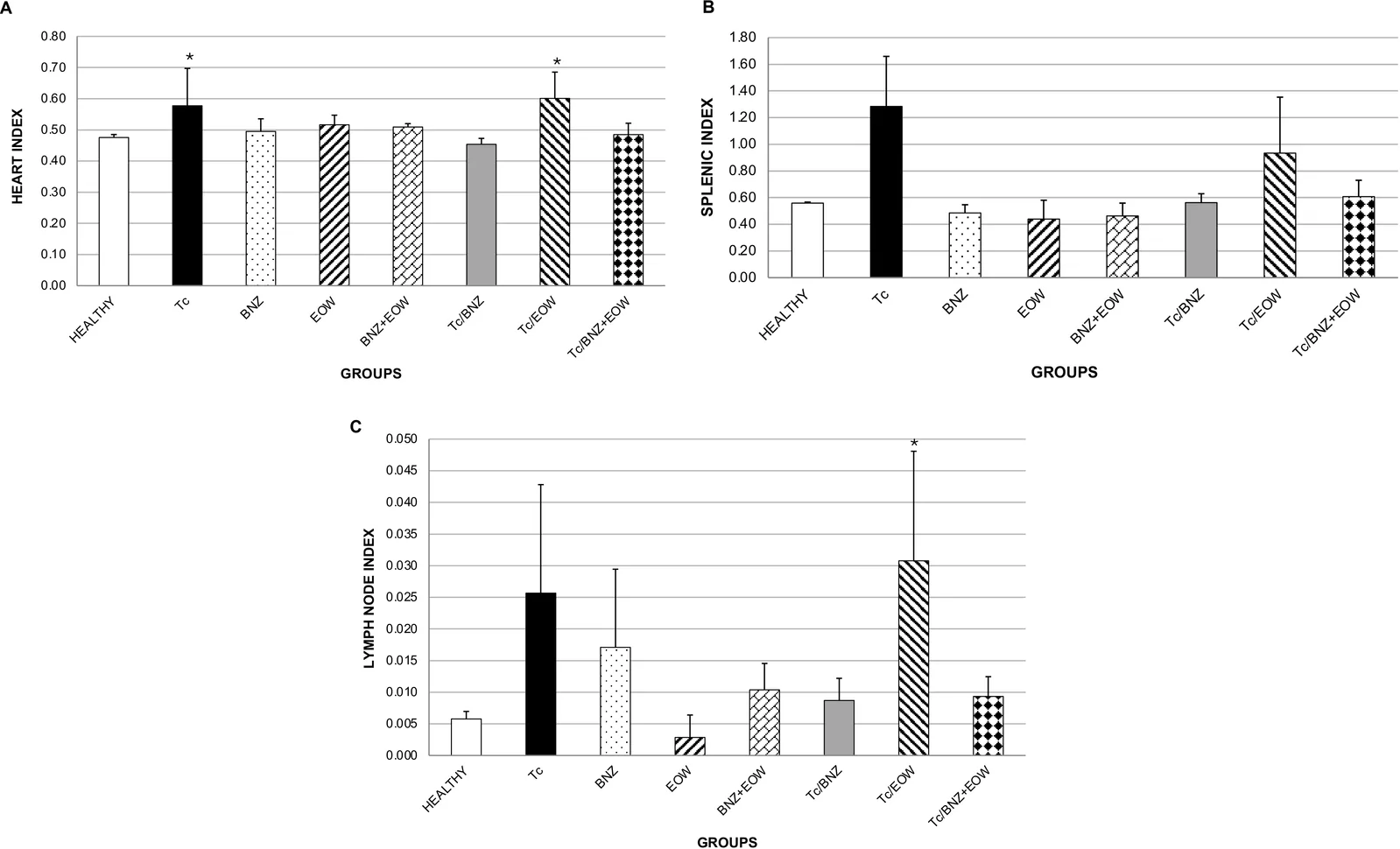
Assessment of organomegaly in a murine model of *Trypanosoma cruzi* infection under treated with benznidazole (BNZ) and electrolyzed oxidizing water (EOW). Data represent the **(A)** heart index, **(B)** splenic index, and **(C)** lymph node index. Evaluations were conducted at 365 days post-infection (dpi), except for the Tc group, where assessments were performed following spontaneous mortality during the acute phase. Each bar represents the mean ± SD. Statistical significance was determined using the Kruskal-Wallis test; differences were considered significant at *p ≤ 0.05 when comparing infected and treated groups against the HEALTHY control group. HEALTHY (uninfected/untreated); Tc (infected/untreated); BNZ, EOW and BNZ+EOW (uninfected mice receiving the respective therapies); Tc/BNZ, Tc/EOW, and Tc/BNZ+EOW (infected mice receiving the respective therapies).

No macroscopic lesions or abnormalities were observed in the collected organs during the necropsies. To assess potential organomegaly, the cardiac, splenic, and lymph node indices were calculated using the formula for organosomatic index. The mean values (± SD) for each experimental group are summarized in **Table 3**. Statistical analysis revealed a significant increase in the cardiac index in both the untreated infected group (Tc) and the Tc/EOW cohort compared to the uninfected healthy control group (**Figure 8A**), indicating infection-induced cardiomegaly. In contrast, evaluation of the splenic index (**Figure 8B**) showed no statistically significant differences across any infected or treated groups relative to healthy controls, indicating the absence of splenomegaly under these experimental conditions. Furthermore, a statistically significant elevation was observed in the lymph node index (**Figure 8C**) specifically in the Tc/EOW group relative to the healthy control, suggesting that EOW monotherapy induced localized lymph node reactivity (lymphadenopathy) in infected animals.

**Table 3:** Organosomatic indices (mean ± SD) of heart, spleen, and lymph nodes in a murine model of *Trypanosoma cruzi* infection treated with benznidazole (BNZ) and electrolyzed oxidizing water (EOW).

Experimental group	Heart index	Spleen index	Lymph nodes index
HEALTHY	0.48 ± 0.0097	0.56 ± 0.0077	0.0058 ± 0.0012
Tc	0.58 ± 0.1198 *	1.28 ± 0.3751	0.0257 ± 0.171
BNZ	0.49 ± 0.0405	0.49 ± 0.0619	0.0171 ± 0.0123
EOW	0.52 ± 0.0308	0.44 ± 0.1415	0.0029 ± 0.0035
BNZ+EOW	0.51 ± 0.0108	0.46 ± 0.0960	0.0104 ± 0.0042
TC/BNZ	0.45 ± 0.0191	0.56 ± 0.0666	0.0087 ± 0.0035
Tc/EOW	0.60 ± 0.0844 *	0.93 ± 0.4194	0.0308 ± 0.0173 *
Tc/BNZ+EOW	0.48 ± 0.0366	0.61 ± 0.1227	0.0093 ± 0.0031

Evaluations were conducted at 365 days post-infection (dpi), except for the Tc group, where measurements were taken following spontaneous mortality during the acute phase. (*) *p* ≤ 0.05 indicates a significant difference relative to the HEALTHY group. HEALTHY: uninfected/untreated control. Tc: *T. cruzi*-infected control. BNZ and EOW: uninfected groups treated with benznidazole or electrolyzed oxidizing water, respectively. BNZ+EOW: uninfected group treated with the combination. Tc/BNZ, Tc/EOW, and Tc/BNZ+EOW: infected groups treated with benznidazole, electrolyzed oxidizing water, or the combination, respectively.

#### 3.6.2 Necropsy: macroscopic findings

Necropsies were performed on BALB/c mice to evaluate macroscopic alterations associated with *T. cruzi* infection and the treatment-induced gross lesions. Systematic inspection of the kidneys, esophagus, and intestinal tract (small and large) revealed no organ damage or morphological changes attributable to either the parasitological infection or the therapeutic compounds.

The kidneys exhibited smooth capsules and preserved external morphology, with no evidence of nephritis, hemorrhage, or fibrosis. The esophageal mucosa appeared homogeneous and intact, without signs of inflammation, ulceration, or wall thickening; notably, no dilatations (megaesophagus) or stenosis were observed. Similarly, the intestinal mucosa remained structurally sound across all experimental groups. Detailed examination of the large intestine showed no evidence of megacolon, mural thickening, or hemorrhagic lesions. These findings suggest that the administered doses of BNZ and EOW were well tolerated under the current experimental conditions, with no evidence of tissue damage, and that the infection did not induce gross systemic pathology in these specific transition tissues (Representative macroscopic images are provided in **Supplementary Figure 2**).

### 3.7 Histopathological findings

To evaluate the therapeutic efficacy of the experimental treatments against tissue damage induced by *T. cruzi*, histopathological analysis was performed on target organs associated with ChD, as well as organs involved in drug metabolism.

**Figure 9:**
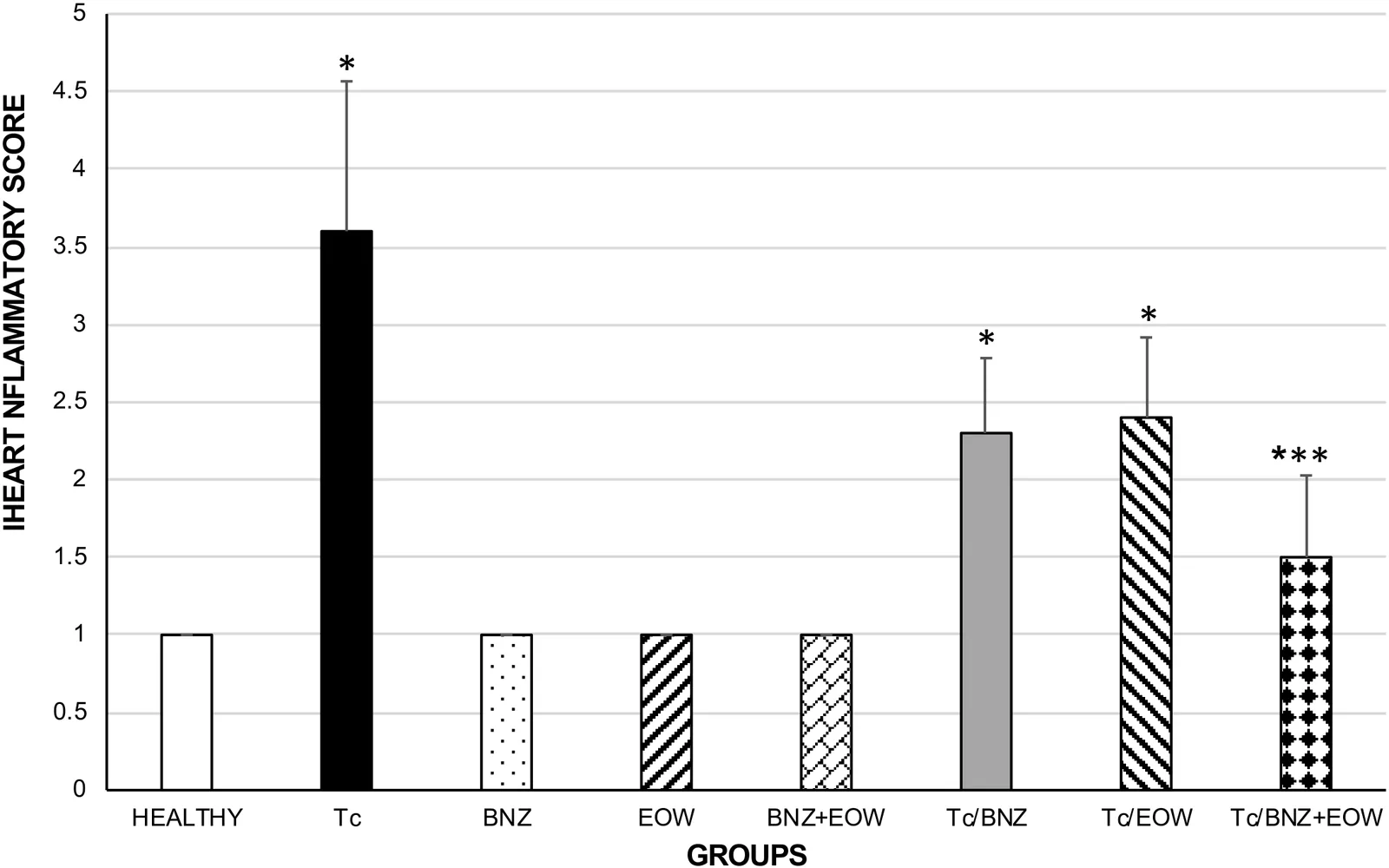
Quantitative histopathological inflammatory scores in cardiac tissue of *T. cruzi*-infected BALB/c mice. The graph illustrates the quantitative severity of lymphocytic infiltration and other alterations across experimental groups evaluated utilizing the standardized 1–5 scoring system detailed in the Materials and Methods section and **Table 2**. Data are expressed as mean ± SD. Multiple group comparisons were performed using the Kruskal-Wallis test; statistically significant differences are explicitly indicated as * *p* ≤ 0.05 when comparing the HEALTHY control group against all other experimental groups, and ***p ≤ 0.05 when comparing the untreated Tc group versus treated groups (BNZ, EOW, and BNZ +EOW).

**Figure 10:**
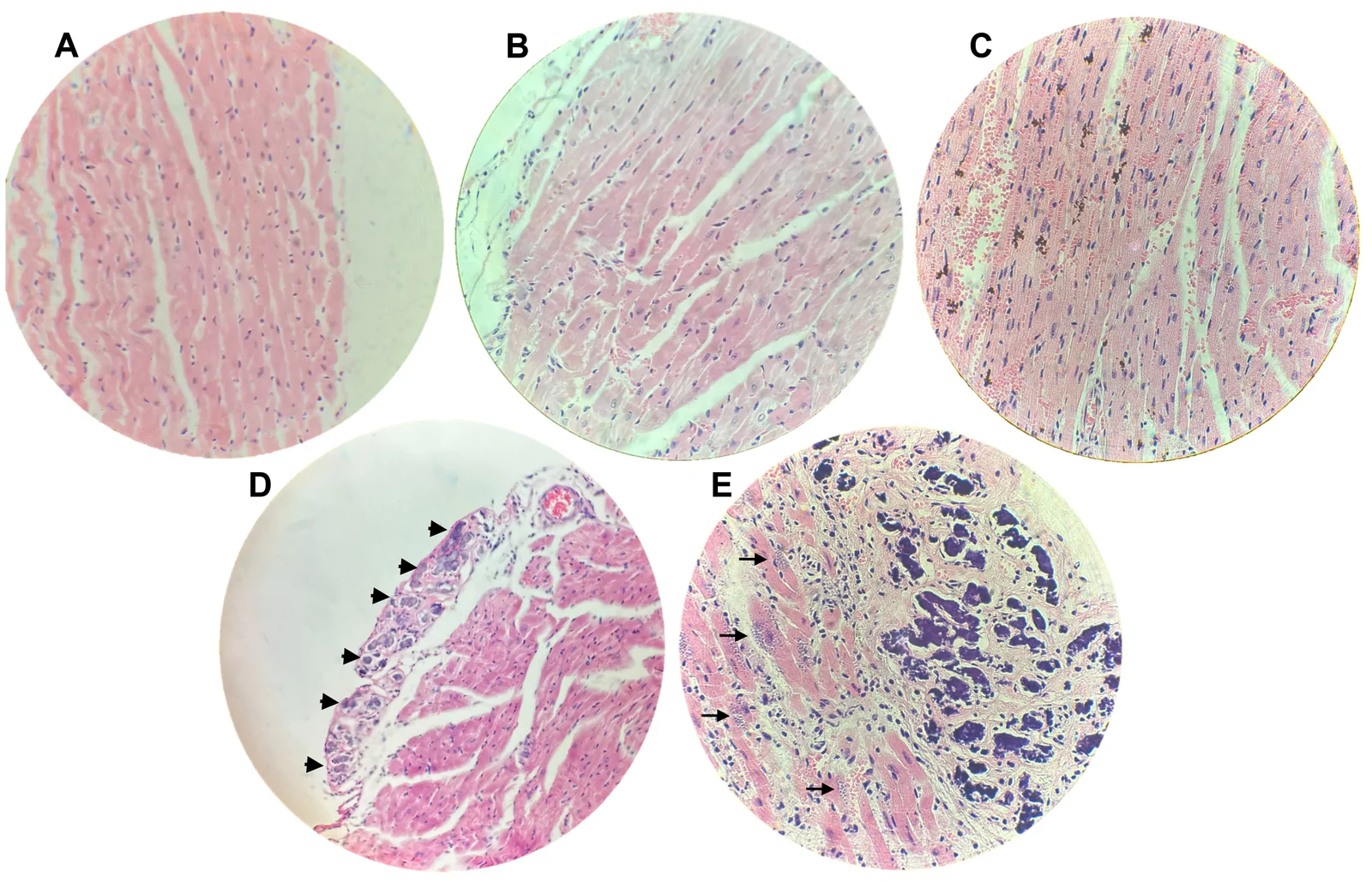
Representative photomicrographs of cardiac tissue sections from *T. cruzi*-infected BALB/c mice. **(A)** Grade 1: Normal myocardial architecture without apparent alterations in the right posterolateral basal region (e.g. HEALTHY group) (40x). **(B)** Grade 2: Mild inflammatory lesions with localized foci of pericarditis and myocarditis in the base of the heart (e.g. Tc/BNZ+EOW group) (40x). **(C)** Grade 3: Moderate pathology showing multiple inflammatory foci in the pericardium and the upper right mediolateral myocardium (e.g. Tc/BNZ) (25x). **(D)** Grade 4: Severe generalized pericarditis accompanied by dystrophic calcifications (black arrowheads) in the mid-right mediolateral region (e.g. Tc group) (25x). **(E)** Grade 5: Critical calcified pericarditis with amastigote nests (black arrows) in the cardiac base (e.g. Tc group) (40x). All sections were stained with Hematoxylin and Eosin (H&E).

In heart sections, chronic pericarditis was observed across all infected groups, with calcified necrosis in some animals from Tc and Tc/EOW groups. However, the Tc group (untreated) exhibited the most severe lesions with 100% of the animals presenting dense lymphocytic myocarditis and interstitial fibrosis prior to mortality (**Figures 9**, **10D, E**). It is critical to note that the untreated Tc group reached 100% mortality during the acute phase, highlighting the high virulence of the H8 strain employed. The inflammatory cell infiltration was also present in groups receiving treatment; nevertheless, the combination of BNZ+EOW proves more effective, resulting in a statistically significant reduction in inflammatory lesion scores (*p* ≤ 0.05) compared to all other treatment regimens (**Figures 9**, **10B**). Specifically, the Tc group consistently reached Grade 5 scores (**Figures 9**, **10E**), characterized by tissue architecture disruption and necrotic foci, whereas the combined treatment successfully maintained scores at Grade 2 (**Figures 9**, **10B**).

The presence of amastigote nests was exclusively detected in the cardiac tissue of the Tc group (**Figures 10E**, arrows). Notably, no parasites were identified in any of the three treatment groups, suggesting that both monotherapies and the combined regimen effectively prevented parasite establishment or facilitated complete clearance from this organ (**Figures 10A–C**).

**Figure 11:**
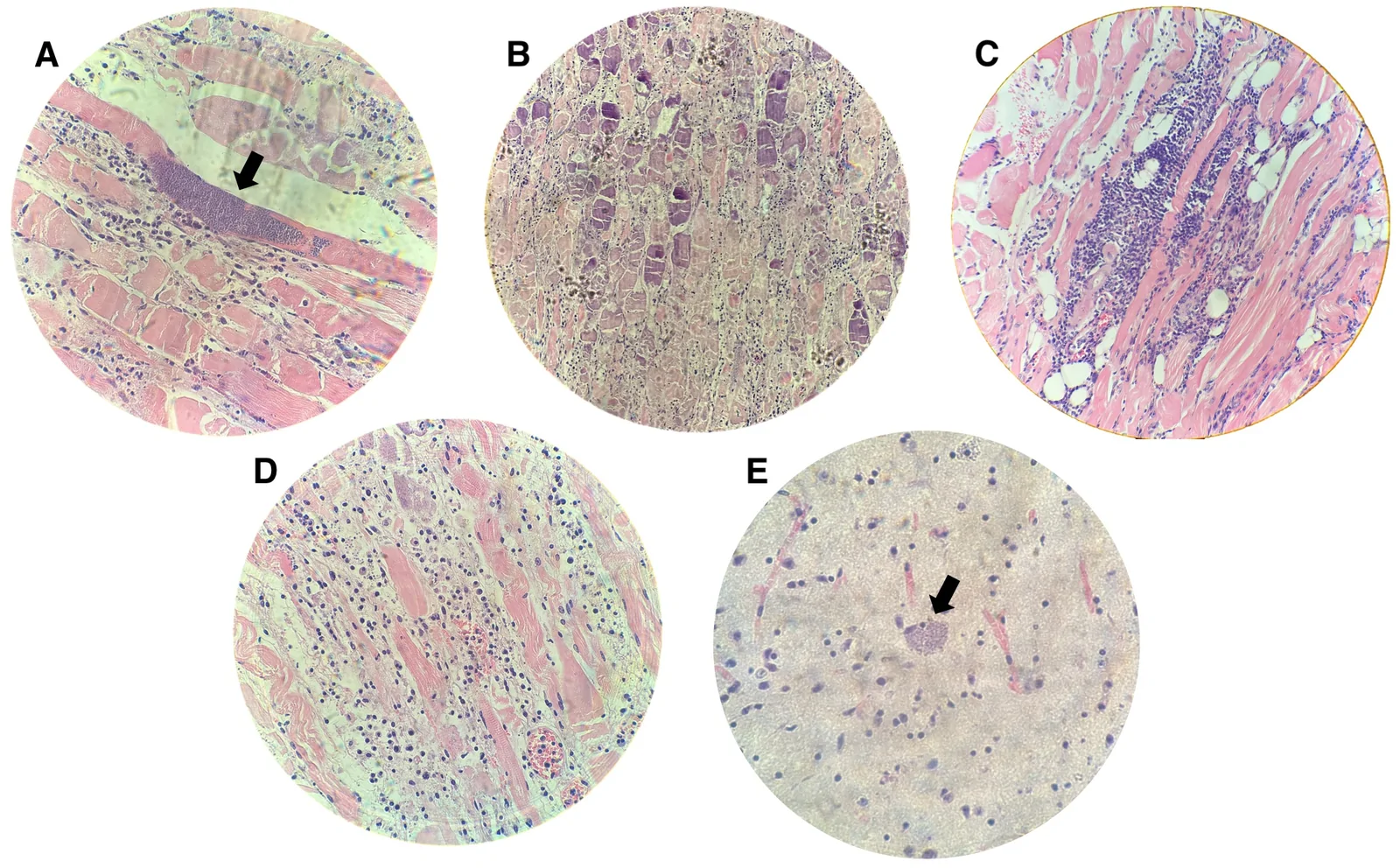
Representative photomicrographs of skeletal muscle and brain sections from *T. cruzi*-infected BALB/c mice. **(A)** Presence of amastigote nests indicated by black arrows in mouse from Tc group (skeletal muscle) (25x); **(B)** areas of moderate necrosis (25x); and **(C)** severe myositis characterized by dense inflammatory infiltration in mouse from Tc group (25x). **(D)** Grade 2 polymorphonuclear leukocyte infiltration in mouse from Tc/BNZ group (skeletal muscle) (40x). **(E)** Identification of an amastigote nest within the brain parenchyma indicated by a black arrow in mouse from Tc/EOW group (40x). All sections were processed with H&E stain.

About extracardiac and systemic histopathology, in the Tc group —where 100% mortality occurred during the acute phase— all individuals (10/10) exhibited moderate to severe inflammation and necrosis, with amastigote nests present in skeletal muscle tissue (**Figures 11A–C**). Additionally, peripheral hepatic infarcts were identified in 20% of these mice (2/10). Regarding the monotherapy groups, the Tc/BNZ group showed predominantly mild (8/10, 80%) and moderate (2/10, 20%) myositis (**Figure 11D**). One individual (10%) from this group presented calcified necrosis involving 5% of the examined muscle tissue (**Figure 11B**). In the Tc/EOW group, mice that succumbed during the acute phase displayed severe polymorphonuclear leukocyte infiltration (Grade 3) and skeletal muscle necrosis (50% of the group); notably, an amastigote nest was identified in the brain of one individual (1/10) (**Figure 11E**), and in mice that reached the chronic stage of this same group, focal necrosis was observed in 5% of the brain tissue in one animal (1/10). For the remaining groups, whose individuals also reached the chronic stage, mild myositis was found in 100% of the infected and treated mice before euthanasia.

Uninfected control groups showed no histopathological alterations in the primary target organs (heart, skeletal muscle, and brain). However, examination of other tissues revealed sporadic findings potentially associated with treatment-related adverse effects. In Group BNZ, chronic interstitial nephritis was identified in 20% of the subjects (2/10). In Group EOW, mild hepatic steatosis was observed in one individual (1/10, 10%). The BNZ+EOW combination group exhibited diffuse ischemic enteritis in 20% of the mice (2/10), along with one case of mild hepatic steatosis (1/10, 10%) (Data not shown).

Additionally, lesions were identified within the digestive tract of infected and treated groups, potentially reflecting an interaction between the *T. cruzi* infection and the pharmacological intervention. In the Tc/BNZ group, 40% of the mice (4/10) showed intestinal involvement, specifically diffuse ischemia of the small intestine (2/10) and chronic peritonitis (2/10). In the Tc/EOW group, one case (1/10) of chronic peritonitis was observed, accompanied by focal myositis of the large intestine. Finally, the Tc/BNZ+EOW group presented a single case (1/10) of mild inflammation in the large intestine (Data not shown).

## 4 Discussion

The conventional BNZ regimen remains controversial due to its significant toxicity and the resulting poor patient adherence in endemic regions. While ongoing clinical trials explore dose-reduction strategies to mitigate these adverse effects, there is a critical need for accessible, cost-effective, and less aggressive therapeutic alternatives for chronic ChD.

Neutral-pH EOW, characterized by its active hypochlorous acid (HOCl) content, has demonstrated broad-spectrum antimicrobial activity at concentrations of 0.1–2.8 mg/mL against bacteria, fungi, and parasites (9, 10). Although oral EOW administration is safe and potentially cytoprotective due to its radical-scavenging properties (18), its therapeutic role in complex parasitic infections remains under scrutiny.

In this study, the high virulence of the *T. cruzi* H8 strain was evidenced by severe weight loss, elevated parasitemia, and 100% mortality in untreated subjects. While BNZ monotherapy remains the most effective intervention for mitigating infection-related decline and restoring weight gain, the BNZ+EOW combination achieved a 99.2% reduction in parasitemia, nearly matching the 100% clearance of the standard drug alone. These findings closely align with previous research on synergistic models, such as the combination of BNZ and voriconazole (19), which frequently encounter challenges like mild toxicity and weight loss *in vivo*, thereby reinforcing BNZ’s standing as the gold standard despite its known pharmacological drawbacks. Furthermore, our data demonstrate that while EOW monotherapy has a moderate independent antiparasitic effect (65.5% reduction), its primary value lies in its high compatibility with standard pharmacological interventions without compromising efficacy.

These findings are consistent with prior research exploring BNZ combination models to control parasitemia and host survival. For instance, Molina Berrios (2012) demonstrated that a high dose of BNZ (100 mg/kg) achieved undetectable parasitemia and 84% survival, while combining aspirin (ASA) with BNZ offered no additional therapeutic advantage over BNZ monotherapy (20). Remarkably, our combination therapy performed similarly to BNZ monotherapy despite our use of a BNZ dosage ten times lower (10 mg/kg). Similarly, Cevey (2018) reported that adding fenofibrate (Fen) to BNZ did not influence parasitemia or cardiac parasite load, as BNZ effectively eliminated *T. cruzi* regardless of the adjunct therapy (21). Regarding host survival, our combination-treated groups exhibited significantly higher survival rates than the infected control group, yet demonstrated equivalent efficacy to the Tc/BNZ group, reinforcing the conclusion that BNZ remains the primary driver of survival and clearance when adjunct therapies are introduced.

A notable discrepancy, however, exists regarding EOW monotherapy: the Tc/EOW group in the present study showed a 50% survival rate, whereas 100% survival under therapeutic administration was observed in our previous work (7). This variation is likely attributable to the higher virulence of the H8 strain used here compared to the Ninoa strain used formerly. Nevertheless, the fact that BNZ+EOW demonstrated similar survival rates to BNZ monotherapy, but superior to EOW alone, suggests that dual-drug management could offer distinct clinical advantages, particularly if it succeeds in reducing overall side effects (22).

Immunologically, EOW has been recognized for its potential to regulate pro-inflammatory cytokines, although its precise mechanism of action remains under investigation (18). In *T. cruzi* infection, the transition from acute to chronic ChD is marked by a dramatic surge in circulating IFN-γ, TNF-α, and IL-6, which typically decrease following successful BNZ treatment (23). During the acute phase (40 dpi), the BNZ+EOW combination triggered a Th1-type pro-inflammatory microenvironment (IgG2a/IgG1 ratio of 4.5), although only BNZ monotherapy reached statistical significance. In the chronic phase, all interventions promoted a more compensated Th1/Th2 profile. While BNZ monotherapy achieved 100% total IgG seronegativity by 365 dpi (contrasting with clinical data from Brazil where a 60-day BNZ course in children achieved only 55.8% negative seroconversion after three years) (24), our BNZ+EOW combination reached a promising 60% seronegativity rate, aligning closely with canine models where EOW-treated animals achieved total negative seroconversion within 12 months (8). In experimental Chagas models, persistent seronegativity serves as the conventional surrogate marker for parasitological cure, mimicking clinical endpoints. While caution is required when extrapolating preclinical findings to human clinical outcomes—such as historical trial data in endemic regions where BNZ regimens achieve partial long-term seroconversion—our findings highlight the robust capacity of this murine model to clear specific humoral responses following sustained therapeutic intervention.

Limiting parasite persistence through effective treatment facilitates a reorganization of the immune system toward a regulatory environment, which is crucial for mitigating chronic Chagas cardiomyopathy (25). While Th1-mediated inflammation is indispensable for acute-phase control, dysregulated inflammation in the chronic stage drives tissue pathogenesis (25, 26). Several researchers have combined BNZ with adjunct agents like ASA, Fen, or pentoxifylline (PTX) to control this exacerbated inflammation; ASA reduces endothelial dysfunction (20), Fen inhibits the nuclear factor kappa B (NF-κB) pathway to regulate pro-fibrotic cytokines (21), and PTX decreases IFN-γ and IL-2 levels to restore homeostasis (25).

Our investigation aligns with these studies, showing that BNZ monotherapy and the BNZ + EOW combination exhibit similar profiles regarding pro-inflammatory cytokine production, with the notable exception of IFN-γ. Additionally, while subtle intra-group variations were detected for IL-2 in the chronic phase, given the lack of an untreated chronic Tc control group (due to 100% acute mortality) and the moderate magnitude of shifts observed, these variations represent statistical trends that warrant cautious interpretation regarding their functional immunological impact. In the chronic stage, IFN-γ levels remained elevated in the combination group—even surpassing the untreated group—but were balanced by high levels of IL-4 (ratio of 1.08), suggesting an intense yet well-modulated response. The differential cytokine production observed in the Tc/BZN+EOW group might be hypothetically explained by the presence of HOCl in the neutral-pH solution. Although not experimentally addressed here, prior literature indicates that HOCl can inhibit signaling pathways associated with the translocation of NF-κB, a master regulator of inflammatory mediators such as IL-1β, IL-6, TNF-α, and transforming growth factor beta (TGF-β) (9, 21).

Intriguingly, we observed a significant chronic elevation of circulating IL-6 levels in infected mice treated with EOW (monotherapy and combination). While sustained IL-6 signaling is often linked to inflammatory pathology, classical IL-6 signaling is indispensable for host survival during *T. cruzi* infection, promoting cell survival microvascular integrity, and skeletal and cardiac muscle regeneration (27). In our model, the elevated chronic levels of IL-6 in EOW-containing regimens did not match with severe tissue damage; conversely, these cohorts displayed a favorable survival rate and only mild, localized miositis with preserved cardiac architecture. This suggests that under EOW treatment, IL-6 may exert a protective, reparative role. Notably, chronic cytokine profiles could not be evaluated for the untreated Tc group due to 100% acute mortality by day 32, making this microenvironment unique to the surviving, treated cohorts where EOW appears to modulate rather than suppresses the inflammatory cascade. Since BNZ monotherapy often fails to sufficiently modulate chronic inflammation (28), adding EOW could enhance the immune response and facilitate a balanced inflammatory profile without compromising antiparasitic efficacy.

Regarding organopathology, the untreated group (Tc) exhibited the most pronounced organomegaly (cardiac, splenic, and lymph node indices), followed by the Tc/EOW cohort. For preventing cardiomegaly, BNZ+EOW offered no advantage over BNZ monotherapy, and the high cardiac index in the Tc/EOW group suggests EOW alone is insufficient to mitigate H8-induced hypertrophy. Splenomegaly and lymphadenopathy remained largely resistant to all interventions, a persistent phenomenon observed with other therapies like nitazoxanide or amphotericin B in murine and canine models (7, 8, 29, 30), underscoring that the visceral inflammatory response is less responsive to therapy than parasitemia.

Histologically, untreated mice exhibited active myocardial amastigote nests, which were notably absent in all treated cohorts (Tc/BNZ, Tc/EOW, and Tc/BNZ+EOW), suggesting effective suppression of intracellular replication. This aligns with findings where BNZ, alone or with Fen, eliminated cardiac parasitism (21), and contrasts with ASA, which lacks independent trypanocidal activity (20). While PTX indirectly influences parasite load through immunomodulation (25), our data reinforce that BNZ remains the primary trypanocidal driver.

The variability of target organ lesions is inherently multifactorial, driven by parasite genetics, inoculum size, inoculation route, and host-strain dynamics (31, 32). A striking finding in this study was the presence of chronic calcified pericarditis in some animals from Tc and Tc/EOW groups. In the BNZ+EOW treatment group, a well-preserved myocardial architecture was observed. In the absence of direct mechanistic evidence, we hypothesize that the immunomodulatory effects of electrolyzed waters (9, 10) may have contributed to this preservation despite the persistent localized infiltrate. Unlike combinations with thioctic acid (TA), which failed to improve clinical outcomes (33), BNZ + EOW shows a nuanced protective profile. The challenge in ChD research remains the discovery of a treatment that balances total parasite elimination with a reduction in chronic, tissue-damaging inflammation—a goal that most immunomodulators and antioxidants have yet to achieve (34). Our research provides compelling evidence for EOW as a therapeutic adjunct; at the histological level, it significantly attenuated inflammatory infiltrates in cardiac and skeletal muscle, providing the physiological basis for increased chronic survival, positioning this combination as a promising, viable therapeutic option for comprehensive ChD management.

While our findings offer promising insights, several limitations must be acknowledged. First, this study utilized a female-only murine model, introducing a potential biological bias due to the known influence of gonadal hormones on *T. cruzi* infection dynamics and therapeutic response (35); future research should incorporate male subjects. Additionally, while the mouse is the gold-standard model for ChD, the translation of these physiological and immunological data to human clinical settings requires caution and further validation in more complex biological systems.

Regarding cardiac damage assessment, the results were less robust than anticipated, suggesting that future experimental designs could benefit from variations in treatment duration or suboptimal BNZ doses to better isolate the adjuvant effect of EOW. Crucially, the inherent and severe mortality associated with *T. cruzi* infection during the acute phase —particularly within the untreated infected (Tc) group— inevitably reduced the effective sample size available for subsequent chronic physiological and immunological evaluations. Although we actively mitigated this biological constraint by pooling the surviving cohorts from two independent experimental blocks (*n* ≤ 10 initial mice per group) and conducting technical duplicates, statistical power for certain chronic endpoint parameters remains limited. Future long-term studies utilizing larger initial cohorts will be essential to fully characterize chronic survival dynamics and long-term immunological outcomes.

Furthermore, interpretation of IgG subclass levels was refined to account for biologically relevant dynamics. Although a *p*-value of 0.05 is the conventional threshold for statistical significance, our findings for IgG2a and IgG1 ratios exhibited a clear statistical trend (0.05 < *p* ≤ 0.1). In *T. cruzi* immunopathology, where subtle shifts in a systemic Th1/Th2 immune microenvironment can critically influence disease progression, adopting a rigid 0.05 cutoff may overlook biologically meaningful immunomodulatory trends.

In addition, our study did not quantify daily food intake or analyze specific digestive and metabolic markers. Consequently, our interpretation of weight fluctuations as potential side effects of the therapies remains hypothetical. Future investigations tracking nutritional intake and metabolic efficiency are warranted to precisely dissect the physiological mechanisms underlying these physical changes.

Finally, although histopathological evidence suggested a favorable physical tolerability of the tested compounds, this study did not include serum biochemical profiles (e.g., transaminases, creatinine, and blood urea nitrogen). While the absence of macroscopic and microscopic tissue damage suggests a lack of potential adverse systemic effects, future studies must incorporate metabolic profiling and clinical chemistry to comprehensively characterize the pharmacological safety of the BZN+EOW combination.

In conclusion, this study demonstrates that the co-administration of BNZ+EOW represents a viable therapeutic approach for ChD models. Our findings show that this combination preserved the essential trypanocidal efficacy of BNZ while incorporating EOW as a complementary adjuvant that assisted in modulating the Th1/Th2 cytokine balance in the chronic phase. Overall, these results highlight the potential of multidrug approach to optimize current therapies by combining parasite clearance with localized immunomodulation.

## Data Availability

The original contributions presented in the study are included in the article/**Supplementary Material**. Further inquiries can be directed to the corresponding author.

## In Memoriam

In memory of Dr. Minerva Arce-Fonseca, who passed away on August 4, 2024. We deeply acknowledge her invaluable scientific supervision in the procedures, and insightful discussion of essential parts of this work. We extend our heartfelt appreciation for her friendship and dedicated hand-in-hand collaboration with Olivia Rodríguez-Morales.
