# Supplementary material for: Combined treatment of benznidazole with an electrolyzed oxidizing water against Trypanosoma cruzi on immunological and anatomopathological features in a murine model of Chagas disease
Source: Front Immunol. 2026 Aug 25;17:1872285. doi: 10.3389/fimmu.2026.1872285 (PMC13549842; doi:10.3389/fimmu.2026.1872285)
Supplement: Supplementary file 1 [file DataSheet1.pdf]

Supplementary material

Table of contents:

Supplementary data	Page
Table S1. IFN-γ/IL-4 ratio.	2
Figure S1. Schematic representation of the experimental workflow and study timeline.	3
Figure S2. Representative macroscopical evaluation during post-mortem examination.	4

Table S1. IFN-γ/IL-4 ratio

IFN-γ / IL-4 ratio			
**Time**	**Group Tc**		
	IFN-γ	IL-4	Ratio
Acute phase	2738.5	318.79	**8.59**
Euth (Chronic phase)	0	0	0

In Tc group, only in acute phase of ChD a polarization to a Th1 or inflammatory response in a 8.59 ratio was observed, as *T. cruzi* infection has always been described at this stage of the disease.

IFN-γ/IL-4 ratio

IFN-γ / IL-4 ratio			
**Time**	**Group Tc/BNZ**		
	IFN-γ	IL-4	Ratio
Acute phase	1335.7083	391.25	**3.41**
Euth (Chronic phase)	1408.25	1610.875	0.87 or 1.14

In Tc/BNZ group, again, only in acute phase of ChD a polarization to a Th1 or inflammatory response in a 3.41 ratio was observed, as *T. cruzi* infection has always been described at this stage of the disease, but more controlled by this reduction of 2.5-fold vs Tc group (8.59).

IFN-γ/IL-4 ratio

IFN-γ / IL-4 ratio			
**Time**	**Group Tc/EOW**		
	IFN-γ	IL-4	Ratio
Acute phase	1907	445.542	**4.28**
Euth (Chronic phase)	3550.0833	5507.833	0.64 or 1.55

In Tc/EOW group, again, only in acute phase of ChD a polarization to a Th1 or inflammatory response in a 4.28 ratio was observed, as *T. cruzi* infection has always been described at this stage of the disease, but more controlled by this reduction of 2.0-fold vs. Tc group (8.59).

IFN-γ/IL-4 ratio

IFN-γ / IL-4 ratio			
**Time**	**Group Tc/BNZ+EOW**		
	IFN-γ	IL-4	Ratio
Acute phase	1975.5417	228.917	**8.63**
Euth (Chronic phase)	7072	6518.25	1.08

In Tc/BNZ+EOW group, only in acute phase of ChD a polarization to a Th1 or inflammatory response in a 8.63 ratio was observed, as *T. cruzi* infection has always been described at this stage of the disease, almost identical compared with Tc group.

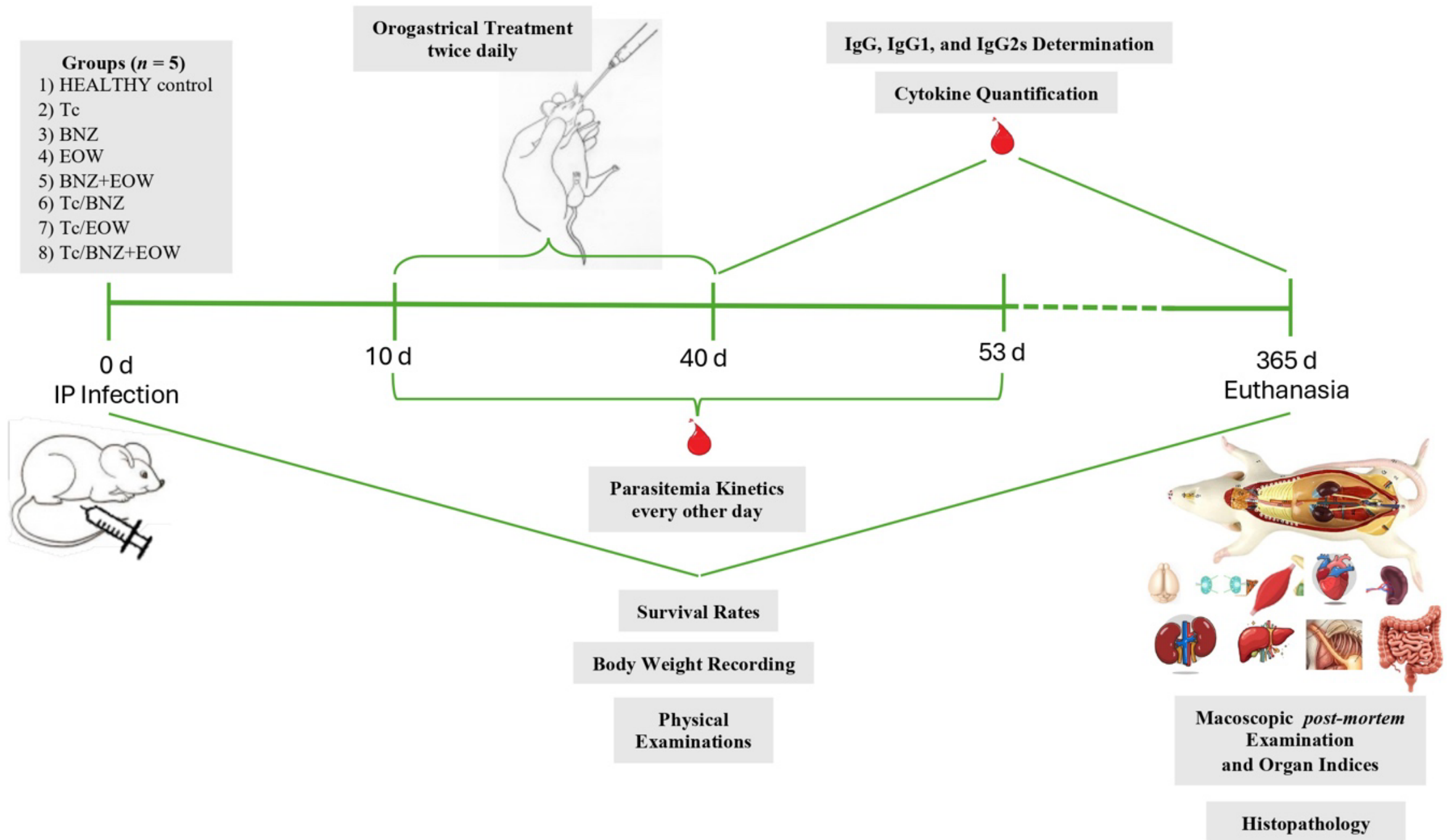

Figure S1. Schematic representation of the experimental workflow and study timeline. The diagram outlines the key methodological stages, including *T. cruzi* infection at day 0 (0 dpi), the treatment regimen period (10-40 dpi; twice daily administration), intermediate blood sampling timepoints, and terminal endpoints (acute at 53 dpi and chronic at 365 dpi euthanasia). HEALTHY: uninfected/untreated control. Tc: *T. cruzi*-infected control. BNZ and EOW: uninfected groups treated with benznidazole or electrolyzed oxidizing water, respectively. BNZ+EOW: uninfected group treated with the combination. Tc/BNZ, Tc/EOW, and Tc/BNZ+EOW: infected groups treated with benznidazole, electrolyzed oxidizing water, or the combination, respectively.

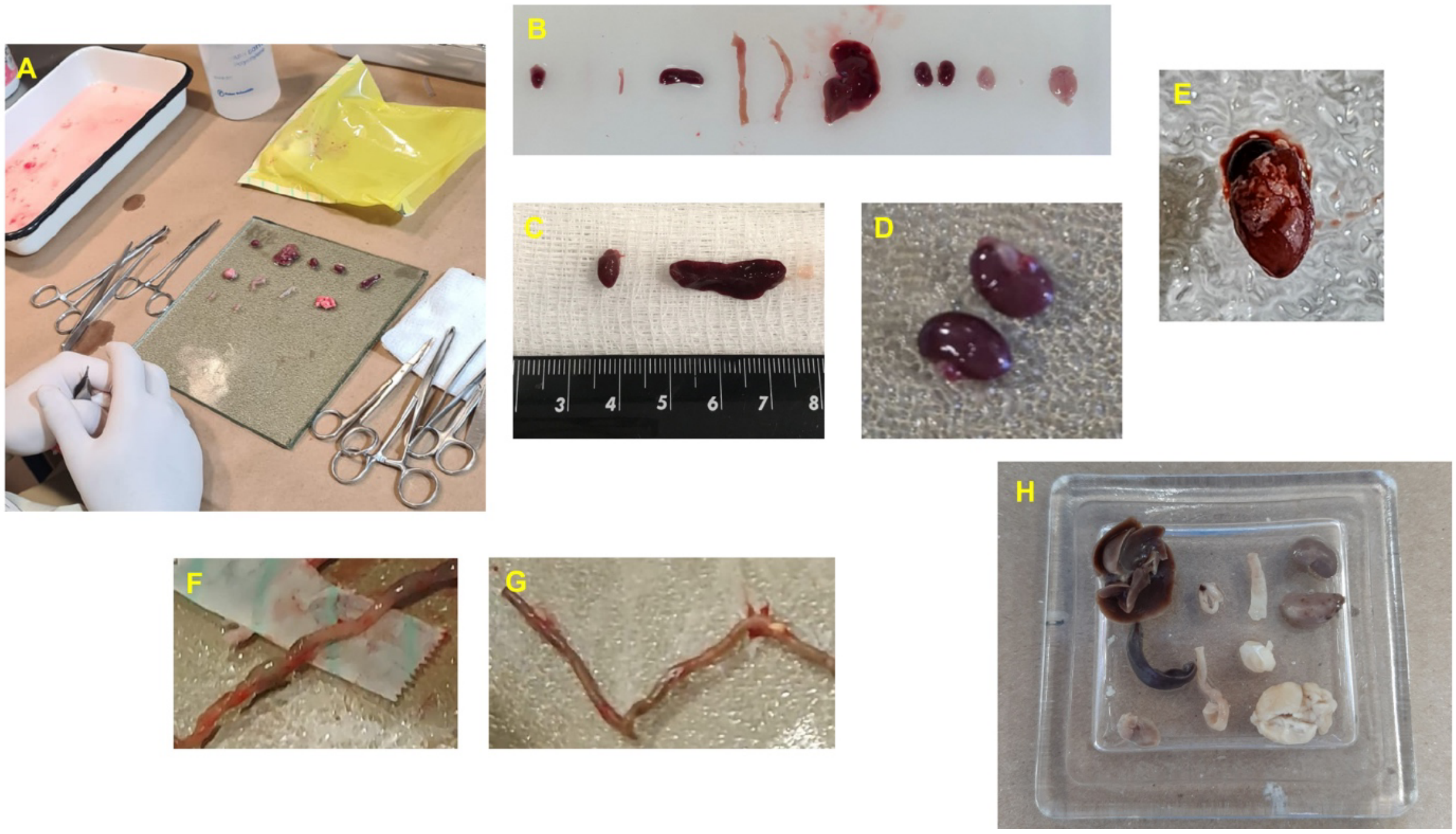

Figure S2. Representative macroscopical evaluation during post-mortem examination. Necropsy was performed to harvest target organs of Chagas disease pathology, as well as tissues to assess the gross tolerability of the therapeutic regimens. Macroscopically, all retrieved organs showed no gross pathological alterations across groups. (A) Surgical workstation, instruments and representative harvested organs. (B) Harvested organs (from left to right) heart, esophagus, spleen, large intestine, small intestine, liver, kidney, skeletal muscle, and brain. (C) Detailed inspection of heart and spleen. (D) Inspection of kidneys. (E) Magnified cardiac examination. (F) Inspection of the large intestine. (G) Inspection of the small intestine. (H) Harvested organs fixed in 10% buffered formalin for subsequent histopathological processing.
